# Impact of single dose of pegfilgrastim on peripheral blood stem cell harvest in patients with multiple myeloma or malignant lymphoma

**DOI:** 10.1038/s41598-025-98453-7

**Published:** 2025-04-25

**Authors:** Hideki Goto, Masashi Sawa, Shin-ichiro Fujiwara, Masaki Ri, Tadao Ishida, Masahiro Takeuchi, Kenji Ishitsuka, Masako Toyosaki, Kazutaka Sunami, Junichi Tsukada, Takashi Sonoki, Aiko Shimogomi, Yuki Ichihashi, Yoshiumi Ouchi, Toshihiro Miyamoto, Masayuki Hino, Yoshinobu Maeda, Takanori Teshima

**Affiliations:** 1 Department of Hematology, Graduate School of Medicine, Hokkaido University Faculty of Medicine, Sapporo, Japan; 2 Division of Laboratory and Transfusion Medicine, Hokkaido University Hospital, Sapporo, Japan; 3 Department of Hematology and Oncology, Anjo Kosei Hospital, Anjo, Japan; 4 Division of Hematology, Department of Medicine, Jichi Medical University, Shimotsuke, Japan; 5 Department of Hematology and Oncology, Graduate School of Medical Sciences, Nagoya City University, Nagoya, Japan; 6 Department of Hematology, Japanese Red Cross Medical Center, Tokyo, Japan; 7 Division of Hematology-Oncology, Chiba Cancer Center, Chiba, Japan; 8 Department of Hematology and Rheumatology, Kagoshima University, Kagoshima, Japan; 9 Department of Hematology and Oncology, School of Medicine, Tokai University, Isehara, Japan; 10 Department of Hematology, NHO Okayama Medical Center, Okayama, Japan; 11 Department of Hematology, University of Occupational and Environmental Health, Kitakyushu, Japan; 12 Department of Hematology/Oncology, Wakayama Medical University, Wakayama, Japan; 13 Kyowa Kirin Co., Ltd, Tokyo, Japan; 14 Department of Hematology, Faculty of Medicine, Institute of Medical Pharmaceutical and Health Sciences, Kanazawa University, Kanazawa, Japan; 15 Department of Hematology, Graduate School of Medicine, Osaka Metropolitan University, Osaka, Japan; 16 Department of Hematology and Oncology, Graduate School of Medicine, Dentistry and Pharmaceutical Sciences, Okayama University, Okayama, Japan

**Keywords:** Multiple myeloma, Pegfilgrastim, Lymphoma, CD34, Mobilization, Peripheral blood stem cell, Myeloma, Clinical trials

## Abstract

This phase 2 study evaluated the impact of pegfilgrastim, a single-dose, long-acting granulocyte colony-stimulating factor, on the steady-state mobilization of hematopoietic stem cells into peripheral blood in patients with multiple myeloma (MM) or malignant lymphoma (ML). Efficacy and safety, along with CD34-positive cell mobilization outcomes were assessed in patients with MM, who were randomly assigned to pegfilgrastim (*n* = 30) or daily filgrastim (*n* = 31), and ML (pegfilgrastim only, *n* = 13) cohorts. In the MM cohort, CD34-positive cell counts ≥ 2 × 10^6^/kg were achieved in 100% of patients in the pegfilgrastim group and 96.7% in the filgrastim group (difference: 3.3%; 80% confidence interval: −0.9–7.5%), demonstrating the non-inferiority of pegfilgrastim to filgrastim. All patients in the ML cohort achieved ≥ 2 × 10^6^/kg CD34-positive cell counts. The plerixafor administration rates in the MM cohort were 50.0% and 63.3% in the pegfilgrastim and filgrastim groups, respectively, and 91.7% in the ML cohort. There were no major differences in safety measures between the two groups. Although the sample size was small, particularly in the ML cohort, a single dose of pegfilgrastim demonstrated comparable efficacy and safety to daily doses of filgrastim, indicating its potential for clinical use while reducing patient burden.

Trial Registration: jRCT2011210029, NCT05007652.

**Supplementary Information:**

The online version contains supplementary material available at 10.1038/s41598-025-98453-7.

## Introduction

Hematopoietic stem cell transplantation (HSCT) is a widely performed medical procedure and a potentially curative therapy for various malignant and non-malignant hematological disorders^1–5^, with autologous HSCT (ASCT) the mainstay of treatment for several hematological diseases. In ASCT, peripheral blood stem cell harvest is the recommended way to mobilize hematopoietic stem cells (HSCs) by using granulocyte colony-stimulating factors (G-CSF) in combination with or without the CXCR4 inhibitor plerixafor. It is well established that a CD34-positive cell count for ASCT of ≥ 2 × 10^6^/kg is a good predictor of successful engraftment and a target for stem cell mobilization^1,6–8^. To achieve this target, a G-CSF such as filgrastim should be administered once or twice daily for approximately 5 days^9^.

Pegfilgrastim is a recombinant human G-CSF, the N-terminus of which is covalently bonded to polyethylene glycol of approximately 20 kDa molecular weight^10^. It has a half-life 10× that of filgrastim and is thus expected to reduce the burden on patients and healthcare providers because it can be administered less frequently^11,12^. Pegfilgrastim is used in daily clinical practice to prevent febrile neutropenia after chemotherapy^13,14^. Previous studies in patients with multiple myeloma (MM) or malignant lymphoma (ML) have reported that administration of pegfilgrastim alone or with plerixafor can mobilize, and increase the harvest of, CD34-positive cells^15–17^. We have also previously reported that low-dose (3.6 mg) pegfilgrastim following cytotoxic chemotherapy increases blood levels of CD34-positive cells^18^. However, the non-inferiority of the peripheral blood stem cell-mobilizing effect of a single dose of pegfilgrastim for ASCT has not been statistically demonstrated or compared with that of conventional, continuous-dose G-CSF.

In this study, we evaluated the effect of a single dose of pegfilgrastim on the steady-state mobilization of HSCs into the peripheral blood in patients with MM or ML. In the MM cohort, the non-inferiority of the mobilization effect of a single subcutaneous administration of pegfilgrastim on HSCs was compared with that from daily subcutaneous administration of filgrastim. The safety, pharmacokinetics, and immunogenicity of pegfilgrastim were also evaluated.

## Methods

### Study design

This multicenter, open-label phase 2 study was conducted in patients with MM or ML at 22 sites in Japan between September 2021 and October 2022. The study included screening, mobilization, and follow-up periods until the safety evaluation (details provided in the **Supplementary Methods**). The study was conducted in accordance with the Declaration of Helsinki and in compliance with the “Act on Securing Quality, Efficacy and Safety of Pharmaceuticals, Medical Devices, Regenerative and Cellular Therapy Products, Gene Therapy Products, and Cosmetics,” and the “Ministerial Ordinance on Good Clinical Practice” (Ministry of Health and Welfare Ordinance No. 28 dated March 27, 1997) and partial amendments. The Institutional Review Board reviewed the eligibility of the study conduct and approved the study based on the study’s ethical, scientific, and medical merits. Written informed consent was obtained from all patients. The study was registered at the Japan Registry for Clinical Trials and ClinicalTrials.gov using the identifiers jRCT2011210029 (06/08/2021) and NCT05007652 (16/08/2021), respectively.

### Patients

Patients aged 20–75 years at the date of informed consent were eligible for the study. The complete inclusion and exclusion criteria are provided in **Supplementary Table ****S1**.

The MM cohort included patients with histologically or pathologically diagnosed MM who achieved partial response or better with induction therapy and intended to undergo ASCT. The ML cohort included patients with histologically or pathologically diagnosed ML who had a first or second complete or partial response. The main exclusion criteria included a diagnosis of malignancies other than, or overlapping with, MM or ML within 2 years before informed consent. Patients were excluded from the MM cohort if they completed chemotherapy within 2 weeks before the first dose of pegfilgrastim or filgrastim. Patients treated with dexamethasone, bortezomib, carfilzomib, ixazomib, or daratumumab could participate, regardless of the treatment timing. For the ML cohort, patients having completed chemotherapy within 2 weeks before pegfilgrastim administration were excluded.

### Intervention

Patients in the MM cohort were randomized 1:1 to receive a single 7.2 mg subcutaneous dose of pegfilgrastim subcutaneously on Day 1–400 µg/m^2^ filgrastim once daily from Day 1 until the end of apheresis. If apheresis was scheduled on the same day as filgrastim dosing, filgrastim was used before the apheresis. Otherwise, there was no limitation on the time of study drug administration. The randomization was conducted using dynamic assignment by the minimization method considering sex and the medical institution as allocation factors through interactive web response system after enrollment by investigators. No blinding was implemented. In the ML cohort, patients were administered 7.2 mg pegfilgrastim as a single subcutaneous dose on Day 1.

Apheresis was performed on Day 5 to collect mobilized HSCs. If the CD34-positive cell count was < 2 × 10^6^/kg, apheresis was not performed on Day 5, or as deemed necessary by the investigator, apheresis could be performed on Day 6 only or on both Days 6 and 7.

If the peripheral blood CD34-positive cell count was ≤ 20/µL on Day 4, plerixafor 0.24 mg/kg could be administered subcutaneously once daily, 9–12 h before apheresis was performed the following day. However, it was acceptable to not administer plerixafor if the leukocyte count was ≥ 50,000/µL. On Days 5 and 6 plerixafor was administered if the total number of CD34-positive cells collected by apheresis was < 2 × 10^6^/kg up to the day of the decision to use plerixafor. However, the drug was not administered if there were safety concerns, such as a leukocyte count ≥ 50,000/µL. Although the total number of CD34-positive cells collected by apheresis was ≥ 2 × 10^6^/kg, plerixafor could be administered at the investigator’s discretion regardless of the above conditions.

### Study endpoints

The primary endpoint was the achievement of ≥ 2 × 10^6^/kg CD34-positive cells collected during the whole apheresis period in the MM cohort. The secondary endpoints were achievement of a CD34-positive cell count ≥ 2 × 10^6^/kg during the whole apheresis period in the ML cohort and changes in CD34-positive cell count in peripheral blood in both cohorts.

The safety endpoints were the incidence of treatment-emergent adverse events (TEAEs) and changes in clinical laboratory values, vital signs, and 12-lead electrocardiogram.

The exploratory endpoints were pharmacokinetics (serum pegfilgrastim concentration), immunogenicity (serum anti-pegfilgrastim antibody levels in the pegfilgrastim group), and TEAEs in the pegfilgrastim group undergoing plerixafor administration.

### Data collection

CD34-positive cells were collected by apheresis and measured using flow cytometry at each institution conducting the trial. A single-platform flow cytometric method was used to determine their count based on the ISHAGE gating strategy^19^.

Serum pegfilgrastim concentration and anti-pegfilgrastim antibodies were measured at LSI Medience Corporation (Tokyo, Japan). Data on TEAEs were collected and classed according to the Common Terminology Criteria for Adverse Events version 5.0.

### Statistical analysis

The sample size in the MM cohort was calculated to show the non-inferiority of pegfilgrastim in the percentage of patients achieving the primary endpoint compared with the filgrastim group. The sample size of the MM cohort was 21 per group to provide a power of 80%, a one-sided significance level of 10%, and a non-inferiority margin of 20%. Considering approximately 20% discontinuation, the target number of patients was set at 27 per group (54 patients in total). Considering the feasibility of the trial, the number of patients for the ML cohort was calculated to be 10.

The definitions of the analytical populations of the study are provided in the **Supplementary Methods**. For the efficacy endpoints, the primary analysis was conducted in the full analysis set. The percentage of achievement of the primary endpoint and its 95% confidence interval (CI) were calculated with the Clopper–Pearson-type CI. Differences between the pegfilgrastim and filgrastim groups in the MM cohort and their 80% and 95% CIs were calculated using Wald-type CIs. Differences between treatment groups and respective 95% CIs for the number of CD34-positive cells in the peripheral blood of the MM cohort by time point of assessment were calculated. For the safety endpoints, obtained data were summarized in the safety analysis set, but no statistical analysis was performed. Additional analysis was conducted using least squares method in the population who received pegfilgrastim.

## Results

### Patient disposition and baseline characteristics

**Fig. 1:**
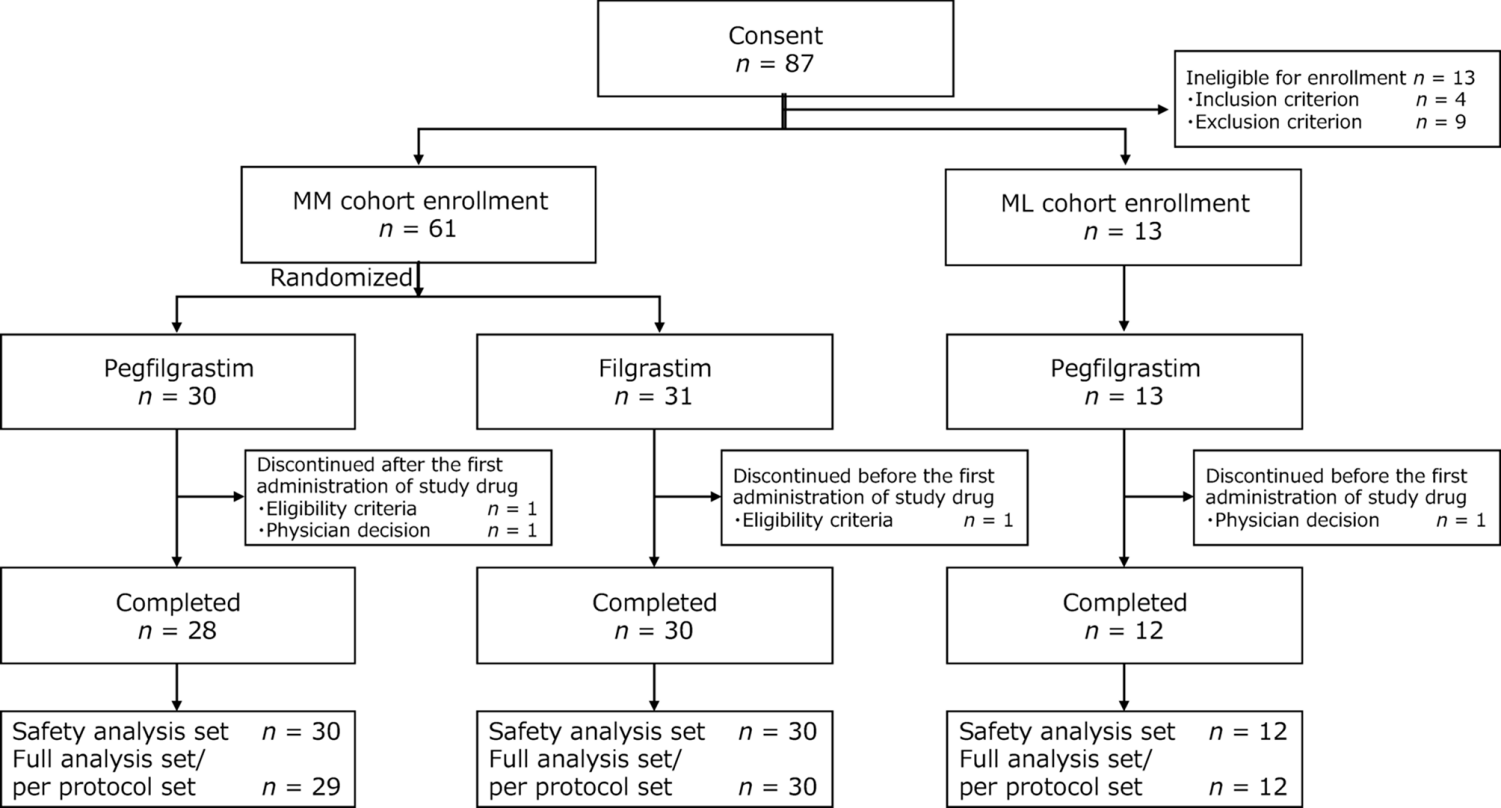
Patient disposition. ML, malignant lymphoma; MM, multiple myeloma.

A total of 87 patients were screened. In the MM cohort, 61 patients were randomly assigned to the pegfilgrastim (*n* = 30) and filgrastim (*n* = 31) groups. Study drug administration and subsequent observation were completed in 28 and 30 patients in the pegfilgrastim and filgrastim groups, respectively. In the ML cohort, 13 patients were enrolled and 12 completed the treatment. Before the first dose of the study drug, one patient in the MM cohort (filgrastim group) was excluded for violation of the eligibility criteria, and one in the ML cohort per the physician’s discretion. After initiating treatment, two patients in the pegfilgrastim group were excluded: one for violation of the eligibility criteria, and one at the physician’s discretion (Fig. 1).

Seventy-two patients (MM cohort: 30 each in the pegfilgrastim and filgrastim groups; ML cohort: 12) were included in the safety analysis set. Seventy-one (MM cohort: 29 in the pegfilgrastim and 30 in the filgrastim group; ML cohort: 12) formed the per protocol and full analysis sets. For the pharmacokinetic and immunogenicity analyses, 42 patients administered pegfilgrastim in both cohorts were included.

In the pegfilgrastim (*n* = 30) and filgrastim (*n* = 30) groups in the MM cohort, patients were predominantly male (19/30 [63.3%] in each group) and the respective median (min–max) ages were 59.0 (38–72) and 60.0 (47–70) years, body weights were 63.4 (36.5–85.0) and 63.6 (38.5–87.1) kg, and CD34-positive cell counts in peripheral blood were 1.0 (0.0–12.6) and 1.0 (0.0–5.0)/µL. Patients in the pegfilgrastim group had received a median (min–max) number of prior therapies of 2.0 (1–3) compared with 2.0 (1–2) in the filgrastim group. There were no major differences in disease-specific and other backgrounds between the two groups (Table 1). In the ML cohort, patients were also predominantly male (*n* = 9; 75.0%), with a median (min–max) age of 63.0 (36–65) years, baseline body weight of 64.4 (48.0–84.0) kg and baseline CD34-positive cell count in peripheral blood of 1.0 (0.0–2.4)/µL (Table 2). Filgrastim was administered until 2 PM in many patients. Especially on the day of apheresis, all patients in filgrastim group received it in the morning.

**Table 1:** Background characteristics of MM patients.

Characteristic	Total *N* = 60	Pegfilgrastim *n* = 30	Filgrastim *n* = 30
Male	38 (63.3)	19 (63.3)	19 (63.3)
Age, years	59.5 (38–72)	59.0 (38–72)	60.0 (47–70)
Body weight, kg	63.6 (36.5–87.1)	63.4 (36.5–85.0)	63.6 (38.5–87.1)
CD34-positive cells in peripheral blood^a^,/µL	1.0 (0.0–12.6)	1.0 (0.0–12.6)	1.0 (0.0–5.0)
Performance status	0 (0–1)	0 (0–1)	0 (0–1)
Body surface area, (m^2^)	1.69 (1.23–2.13)	1.65 (1.25–2.01)	1.69 (1.23–2.13)
Number of prior therapies	2.0 (1–3)	2.0 (1–3)	2.0 (1–2)
Previous immunomodulatory drug	55 (91.7)	29 (96.7)	26 (86.7)
Lenalidomide	54 (90.0)	29 (96.7)	25 (83.3)
Pomalidomide	3 (5.0)	2 (6.7)	1 (3.3)
Previous anti-CD38 antibody	13 (21.7)	7 (23.3)	6 (20.0)
Daratumumab	12 (20.0)	6 (20.0)	6 (20.0)
Isatuximab	1 (1.7)	1 (3.3)	0 (0.0)
Previous proteasome inhibitor	58 (96.7)	29 (96.7)	29 (96.7)
Bortezomib	58 (96.7)	29 (96.7)	29 (96.7)
Carfilzomib	9 (15.0)	5 (16.7)	4 (13.3)
Prior radiation therapy	3 (5.0)	1 (3.3)	2 (6.7)
Stage			
I	19 (31.7)	11 (36.7)	8 (26.7)
II	24 (40.0)	11 (36.7)	13 (43.3)
III	17 (28.3)	8 (26.7)	9 (30.0)

Data are n (%) or median (min–max). MM, multiple myeloma. ^a^CD34-positive cells at baseline were collected on Day 1 or on the day before Day 1, prior to the administration of the study drug.

**Table 2:** Background characteristics of ML patients.

Characteristic	Pegfilgrastim *n* = 12
Male	9 (75.0)
Age, years	63.0 (36–65)
Body weight, kg	64.4 (48.0–84.0)
CD34-positive cells in peripheral blood^a^,/µL	1.0 (0.0–2.4)
Performance status	0 (0–1)
Body surface area, (m^2^)	1.76 (1.43–2.08)
Number of prior therapies	2.0 (1–5)
Prior systemic therapy	
R-CHOP	8 (66.7)
BR	2 (16.7)
R-GDP	2 (16.7)
Brentuximab vedotin (including combination therapy)	2 (16.7)
R-DA-EPOCH	1 (8.3)
Prior radiation therapy	2 (16.7)
Stage	
I	2 (16.7)
II	3 (25.0)
III	1 (8.3)
IV	5 (41.7)
Not applicable	1 (8.3)
Subtype	
Mantle cell	3 (25.0)
Diffuse large B-cell, not otherwise specified	7 (58.3)
Peripheral T-cell	1 (8.3)
Hodgkin	1 (8.3)

Data are n (%) or median (min–max). ^a^CD34-positive cells at baseline were collected on Day 1 or on the day before Day 1, prior to the administration of the study drug. ML, malignant lymphoma; R-CHOP, rituximab + cyclophosphamide + doxorubicin + vincristine + prednisolone; BR, bendamustine + rituximab; R-GDP, rituximab + dexamethasone + gemcitabine + cisplatin; R-DA-EPOCH, rituximab + etoposide + doxorubicin + vincristine + cyclophosphamide + prednisolone.

### MM cohort

#### Efficacy

On Day 5, the proportions of patients achieving CD34-positive cell counts ≥ 2 × 10^6^/kg while receiving pegfilgrastim and filgrastim were 86.2% (25/29 patients) and 96.7% (29/30 patients), respectively (Table 3). With pegfilgrastim, the proportion was 100% (29/29 patients) through Days 5–6, while it remained at 96.7% (29/30 patients) with filgrastim. In all cases, the first apheresis was conducted on Day 5 with none conducted on Day 7. The difference in the numbers of patients achieving CD34-positive cell counts ≥ 2 × 10^6^/kg between the treatment groups (pegfilgrastim − filgrastim) was 3.3% (two-sided 80% CI − 0.9%−7.5%). The lower limit of the two-sided 80% CI for the difference was − 0.9%, which is higher than the non-inferiority margin of − 20%. Therefore, the non-inferiority of pegfilgrastim to filgrastim was demonstrated.

**Table 3:** Achievement of CD34-positive cells ≥ 2 × 10^6^/kg.

Day	MM Cohort							ML Cohort		
	Pegfilgrastim *n* = 29		Filgrastim *n* = 30		Difference			Pegfilgrastim *n* = 12		
	*n* (%)	95% CI	*n* (%)	95% CI		80% CI	95% CI	*n* (%)		95% CI
5	25 (86.2)	(68.3–96.1)	29 (96.7)	(82.8–99.9)	−10.5		(− 24.6–3.6)	8 (66.7)		(34.9–90.1)
5–6	29 (100.0)	(88.1–100)	29 (96.7)	(82.8–99.9)	3.3	(− 0.9–7.5)	(− 3.1–9.8)	12 (100)		(73.5–100)

CI, confidence interval; ML, malignant lymphoma; MM, multiple myeloma.

The prior systemic therapies of the patient who did not achieve the threshold in the filgrastim group were bortezomib + cyclophosphamide + dexamethasone and daratumumab + lenalidomide + dexamethasone. The administration rates of plerixafor were 50.0% (15/30 patients) in the pegfilgrastim group and 63.3% (19/30 patients) in the filgrastim group. No patient received additional plerixafor if the number of CD34-positive cells collected exceeded 2 × 10^6^/kg.

**Supplementary Fig. ****S1** and **Supplementary Table S2**, respectively, show the mean and median CD34-positive cell counts per day during apheresis. The mean ± standard deviation (SD) cell count was 4.97 ± 3.17 × 10^6^/kg in the pegfilgrastim group while that in the filgrastim group was 4.95 ± 2.42 × 10^6^/kg on Day 5. Among several patients who underwent second apheresis on Day 6, the mean ± SD cell counts were 2.38 ± 0.88 × 10^6^/kg and 0.93 ± 0.37 × 10^6^/kg in the pegfilgrastim and filgrastim groups, respectively.

**Fig. 2:**
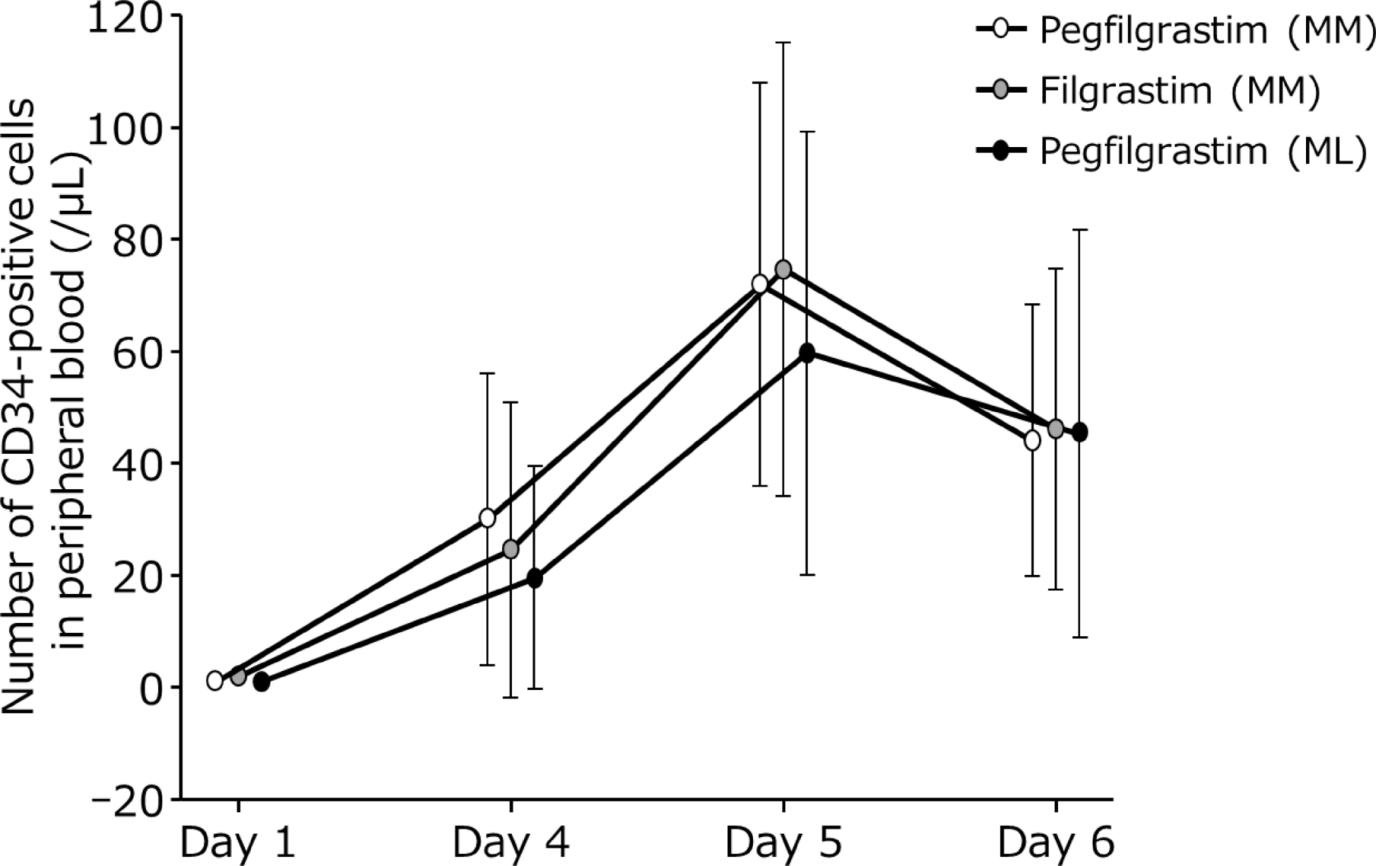
Changes in CD34-positive cell count in peripheral blood. Error bars indicate standard deviation. ML, malignant lymphoma; MM, multiple myeloma.

Changes in CD34-positive cell count in peripheral blood in each cohort are shown in Fig. 2 and **Supplementary Table S3**. After study drug administration in the pegfilgrastim and filgrastim groups, the median (min–max) number of CD34-positive cells in peripheral blood increased over time, reaching 22.7 (3.70–90.0)/µL and 14.5 (1.30–101)/µL on Day 4 and a maximum of 68.8 (12.0–138)/µL and 72.0 (6.00–171)/µL on Day 5, respectively. Thereafter, the number of CD34-positive cells decreased on Day 6 to 37.9 (9.20–108)/µL and 40.2 (5.50–140)/µL in the pegfilgrastim and filgrastim groups, respectively.

In patients who did not achieve CD34-positive cell counts ≥ 2 × 10^6^/kg in a single apheresis, the median (min–max) CD34-positive cell counts in peripheral blood just before collection was 25.7 (6.00–116)/µL (**Supplementary Table S4**). Four of these patients had CD34-positive cell counts of < 20/µL. Of the people who had received a bortezomib + lenalidomide + dexamethasone regimen before, the median (min–max) peripheral blood CD34-positive cell counts were 69.4 (12.0–138)/µL in the pegfilgrastim group and 76.6 (11.3–171)/µL in the filgrastim group.

The mobilization effect was moderately negatively correlated with patient age (*r* = − 0.526), but there was no correlation with body weight (*r* = − 0.053) or body surface area (*r* = − 0.045) (**Supplementary Fig. S2**).

#### Safety

TEAEs occurred in 24/30 patients (80.0%) in each of the two groups (Table 4). One Grade ≥ 3 TEAE, hypocalcemia, occurred in the pegfilgrastim group and had no causal relationship to pegfilgrastim (**Supplementary Table S5**). Of TEAEs that occurred in > 10% of patients in the pegfilgrastim group, alkaline phosphatase elevation (26.7%), headache (20.0%), and diarrhea (10.0%) tended to occur more frequently than in the filgrastim group. Grade 1 interstitial lung disease occurred in one patient (3.3%) in the pegfilgrastim group and was determined to have a causal relationship with the study drug. The patient recovered without therapeutic intervention, but follow-up was terminated at the discretion of the physician in charge.

**Table 4:** Most common TEAEs (with a frequency of ≥ 10% in patients receiving pegfilgrastim).

	MM cohort			ML cohort	Pegfilgrastim total
	Total *N* = 60	Pegfilgrastim *n* = 30	Filgrastim *n* = 30	Pegfilgrastim *n* = 12	*n* = 42
	*n* (%)	*n* (%)	*n* (%)	*n* (%)	*n* (%)
Patients with any TEAE	48 (80.0)	24 (80.0)	24 (80.0)	10 (83.3)	34 (81.0)
Back pain	15 (25.0)	9 (30.0)	6 (20.0)	2 (16.7)	11 (26.2)
Lactate dehydrogenase increased	11 (18.3)	7 (23.3)	4 (13.3)	1 (8.3)	8 (19.0)
Alkaline phosphatase increased	10 (16.7)	8 (26.7)	2 (6.7)	0	8 (19.0)
Headache	8 (13.3)	6 (20.0)	2 (6.7)	1 (8.3)	7 (16.7)
Diarrhea	4 (6.7)	3 (10.0)	1 (3.3)	4 (33.3)	7 (16.7)
Hyperuricemia	7 (11.7)	5 (16.7)	2 (6.7)	0	5 (11.9)
Pyrexia	7 (11.7)	4 (13.3)	3 (10.0)	1 (8.3)	5 (11.9)
Bone pain	6 (10.0)	3 (10.0)	3 (10.0)	2 (16.7)	5 (11.9)

ML, malignant lymphoma; MM, multiple myeloma; TEAE, treatment-emergent adverse event.

### ML cohort

#### Efficacy

The proportion of patients achieving CD34-positive cell counts ≥ 2 × 10^6^/kg was 66.7% on Day 5, but on Days 5–6, 12/12 (100%; 95% CI: 73.5%, 100%) patients had ≥ 2 × 10^6^/kg CD34-positive cells (Table 3). On Days 5 and 6, 3.92 ± 2.11 × 10^6^/kg and 1.95 ± 1.63 × 10^6^/kg cells, respectively, were collected. After study drug administration, the median (min–max) number of CD34-positive cells in peripheral blood increased over time, reaching 14.4 (5.30–79.4)/µL on Day 4, peaking at 49.2 (10.0–151)/µL on Day 5, and decreasing to 29.8 (13.8–134)/µL on Day 6. Plerixafor was administered to 91.7% of patients (**Supplementary Table S2**).

#### Safety

TEAEs occurred in 10/12 patients (83.3%) (Table 4). One serious TEAE, right peroneal neuritis, occurred in the filgrastim-treated group but was determined to have no causal relationship with the study drug.

Grade 1 hepatosplenomegaly occurred in one patient (3.3%) in the pegfilgrastim-treated group, and was determined to have a causal relationship with the study drug. The patient did not recover during the follow-up period, and follow-up was terminated at the discretion of the physician in charge.

### General safety findings

**Fig. 3:**
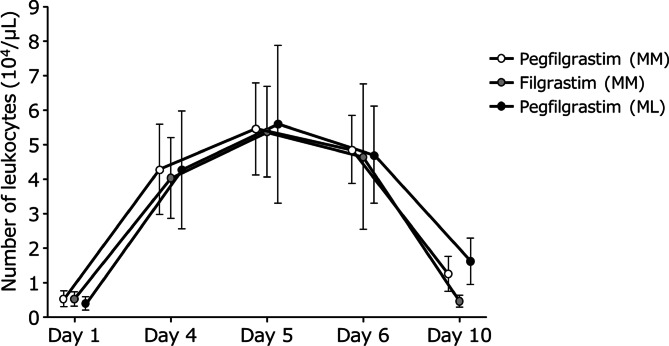
Changes in leukocyte count. Error bars indicate standard deviation. ML, malignant lymphoma; MM, multiple myeloma.

Leukocyte count increases occurred in the pegfilgrastim and filgrastim groups from ˂10,000/µL to ≥ 40,000/µL on Day 4, reached a maximum level of ˂60,000/µL on Day 5, and decreased progressively thereafter, reaching baseline levels by Day 10 (Fig. 3). No other significant changes in laboratory values and vital signs were observed (**Supplementary Table S6**). No adverse events resulted in death or other significant events in either group.

### Exploratory endpoints

#### Pharmacokinetics and immunogenicity

Day 4 mean ± SD serum pegfilgrastim concentrations after single subcutaneous administrations of 7.2 mg pegfilgrastim to MM (*n* = 27) and ML (*n* = 12) patients were 130 ± 142 ng/mL and 139 ± 142 ng/mL, respectively. There were no major differences between the two groups. No patients tested positive for anti-pegfilgrastim binding antibodies after pegfilgrastim administration.

#### Safety with concomitant pegfilgrastim and plerixafor administration

Of the patients in the MM cohort treated with plerixafor, 15/15 (100%) in the pegfilgrastim group and 18/19 patients (94.7%) in the filgrastim group achieved a CD34-positive cell count ≥ 2 × 10^6^/kg, compared with 14/14 (100%) and 11/11 patients (100%), respectively, in those who did not receive plerixafor concomitantly before the achievement of the primary endpoint. Overall safety was comparable between the patients with plerixafor and without it across each treatment group (**Supplementary Table S7**).

## Discussion

HSC mobilization has long been implemented in combination with chemotherapy such as cyclophosphamide. In recent studies, steady-state HSC mobilization with G-CSF was considered to be as effective as and less toxic than a chemotherapy combination^20–22^. Several studies showed the efficacy of pegfilgrastim with single-arm, or at the most with comparative daily-dose G-CSF, in small cohorts^15–17^. In this phase 2, prospective, randomized, and parallel trial, the non-inferiority of the efficacy of single-dose vs. daily-dose G-CSF was statistically demonstrated. All patients in the pegfilgrastim group of the MM cohort achieved CD34-positive cell counts > 2 × 10^6^/kg, a widely accepted target for HSCT, and 96.7% in the filgrastim group. Achievement of this target was 100% in the ML cohort. In most patients with MM and more than half of patients with ML, sufficient HSCs could be collected in a single apheresis on Day 5. This indicates that the timing of apheresis could be fixed by monitoring HSCs in peripheral blood. It is unclear why one patient failed to mobilize sufficient CD34-positive cells. However, they had received heavy prior systemic therapies, which might have led to mobilization failure.

HSC mobilization with filgrastim requires daily dosing because of its short half-life; thus, an option to reduce the dosing frequency to reduce patient burden is desirable. Numerous studies show that the ability to harvest > 2 × 10^6^/kg HSCs is a factor for favorable transplant outcomes^18,19,23–26^. In this study, as with conventional G-CSF^9^, the target value of 2 × 10^6^/kg was achieved in all patients in the pegfilgrastim group within one or two cycles of apheresis. MM patients usually need > 4 × 10^6^/kg HSCs to conduct tandem HSCTs in daily clinical settings. In the MM cohort in this study, 62.0% and 66.7% in the pegfilgrastim group and the filgrastim group achieved > 4 × 10^6^/kg, while the rest did not. There were some possible factors explaining why this target was not reached, but the most likely relates to the target cell count. In this study, the target cell count was 2 × 10^6^/kg; whether more cycles of apheresis were conducted depended on the policy in each institution. Therefore, it may be that not all patients who achieved the target of > 4 × 10^6^/kg had samples collected. However, considering that the median number of CD34-positive cells just before the first apheresis in the MM cohort was 68.8 cells/µL with pegfilgrastim and 72.0 cells/µL with filgrastim, the results were well applicable for the patients who target > 4 × 10^6^/kg of HSCs^7,27^.

There was very little difference between the pegfilgrastim and filgrastim groups in the number of peripheral blood CD34-positive cells collected. Peripheral blood CD34-positive cell counts peaked on Day 5 in both cohorts, with more marked changes in the MM cohort. CD34-positive cells decreased after Day 6 probably because of the apheresis performed on Day 5. In the pegfilgrastim groups in both cohorts, moderate negative correlations between mobilization effect and age were observed, with no relationship between mobilization effect and body weight or body surface area. These results are consistent with a previous report investigating lymphoma, which suggested that the younger the patient was, the more CD34-positive cells were mobilized^18^. Patients who previously received the bortezomib + lenalidomide + dexamethasone regimen, which is commonly used as induction therapy for transplant eligible patients, had CD34-positive cells well mobilized to peripheral blood in both the pegfilgrastim and filgrastim groups.

In this study, a single subcutaneous administration of pegfilgrastim resulted in efficacy and safety parameters similar to those of daily subcutaneous administration of filgrastim. It has been reported that pegfilgrastim can be used to mobilize HSCs in patients indicated for ML transplantation^17,28,29^. This is consistent with other phase 2 studies in which pegfilgrastim was highly effective for mobilizing CD34-positive cells in pretreated ML or MM patients^30,31^.

Overall, in this study, pegfilgrastim was well tolerated in MM and ML patients, with similar adverse event profiles as the filgrastim group. Only one Grade 3 TEAE (hypocalcemia) occurred in the pegfilgrastim-treated group, but there was no relationship to pegfilgrastim. The safety profile for pegfilgrastim in this study was consistent with previous reports^32^, including confirmation of the safety and HSC mobilization effect of 7.2 mg pegfilgrastim in healthy adults^33^.

No patients tested positive for anti-pegfilgrastim binding antibodies after pegfilgrastim administration and no differences were observed in pharmacokinetic parameters, suggesting that it is unlikely that anti-drug antibody status affected efficacy or safety in this study.

The approved dose for the prevention of febrile neutropenia is 3.6 mg in Japan^34^, while the approved dose worldwide is 6 mg^35^. Therefore, the dose of pegfilgrastim used in this study (7.2 mg) was lower than the dose (12 mg) that has been verified for stem cell mobilization worldwide^15,28,36^. Additional studies are needed to determine whether the appropriate dose varies by population.

Another relevant finding in our study was that approximately 60% of patients in the MM cohort and over 90% of patients in the ML cohort received the chemokine receptor antagonist plerixafor as a concomitant drug, and its safety in combination with pegfilgrastim was acceptable. Previous studies, including in patients with MM, have reported significantly higher numbers of HSCs collected when G-CSF preparations were combined with plerixafor compared with mobilization with G-CSF alone^37–39^. Our finding in the MM cohort, that all the patients achieved the target (≥ 2 × 10^6^/kg) while the rate of plerixafor administration was < 60%, indicates that pegfilgrastim alone can mobilize and allow the collection of sufficient HSCs from peripheral blood if patients have sufficient CD34-positive cells in peripheral blood before apheresis. Even in patients whose CD34-positive cells were < 20/µL, pegfilgrastim with plerixafor mobilized sufficient HSCs with acceptable safety.

In terms of cost-effectiveness, although pegfilgrastim is more expensive than conventional daily G-CSF, it may be a useful option for HSC mobilization because it offers significant advantages to both providers and patients by reducing treatment frequency, eliminating daily blood cell monitoring, and reducing the discomfort of receiving injections. Another significant advantage for healthcare providers is that, unlike filgrastim, pegfilgrastim has a fixed dose for administration, reducing the time and effort required for preparation. As for the use of plerixafor, its high cost is well known in HSCs mobilization, but in many clinical trials including the pivotal phase 3 trials^37,38,40–42^, the use and dosage of plerixafor was fixed for mobilizing HSCs regardless of patients’ condition. Several studies reported that flexible administration of plerixafor use led to sufficient amounts of collected HSCs and similar results were obtained in this trial^43–45^. This indicates that the administration of plerixafor could have the advantage of reducing the number of collection times even if combined with pegfilgrastim.

Our study had certain limitations, including the small sample size, that may indicate insufficient generalizability of the findings. The target number of HSCs was different depending on the policy of each facility, which may have caused a bias in the number of CD34-positive cells collected. We used an 80% CI to demonstrate non-inferiority, which may have increased the risk of false positives. Additionally, no direct comparison of pegfilgrastim and filgrastim was made in patients with ML. Although the results of pegfilgrastim in ML were comparable to those of filgrastim in previous reports, a larger number of cases, particularly for ML, needs to be analyzed to confirm the findings in this study. Future studies should also investigate the appropriate dosage of pegfilgrastim and the administration criteria for plerixafor.

## Conclusion

The findings of this phase 2, prospective, randomized trial suggest that in patients with MM, pegfilgrastim treatment was as effective as filgrastim in mobilizing HSCs. Although the sample size of the ML cohort was small, and there was no direct comparison with filgrastim, pegfilgrastim demonstrated efficacy for HSC mobilization in these patients. No serious TEAEs occurred during pegfilgrastim treatment, suggesting it may be a good option for mobilizing HSCs, with benefits for both healthcare providers and patients because of the extended dosing interval and fixed collection date. The results also suggest that the administration of plerixafor could be adjusted according to the number of CD34 cells in the peripheral blood on the day before collection. The use of pegfilgrastim in HSC harvesting from peripheral blood may reduce the burden on patients and medical institutions compared to conventional G-CSF, resulting in benefits for clinical practice.

## Electronic supplementary material

Below is the link to the electronic supplementary material.

Supplementary Material 1

## Data Availability

The datasets generated and/or analyzed will be available in the Vivli repository, https://vivli.org/ourmember/kyowa-kirin/, as long as conditions of data disclosure specified in the policy section of the Vivli website are satisfied.

## References

[1] Duong, H. K. et al. Peripheral blood progenitor cell mobilization for autologous and allogeneic hematopoietic cell transplantation: guidelines from the American society for blood and marrow transplantation. *Biol. Blood Marrow Transpl.* **20**, 1262–1273 (2014). 10.1016/j.bbmt.2014.05.003 24816581

[2] Palumbo, A. et al. Autologous transplantation and maintenance therapy in multiple myeloma. *N Engl. J. Med.* **371**, 895–905 (2014). 25184862 10.1056/NEJMoa1402888

[3] Wang, J. et al. Comparison of survival between autologous and allogeneic stem cell transplantation in patients with relapsed or refractory b-cell non-Hodgkin lymphoma: a meta-analysis. *Cell. Transpl.* **29**, 963689720975397 (2020). 10.1177/0963689720975397 PMC7784574 33238731

[4] Cornelissen, J. J. & Blaise, D. Hematopoietic stem cell transplantation for patients with AML in first complete remission. *Blood* **127**, 62–70 (2016). 26660427 10.1182/blood-2015-07-604546

[5] Kröger, N. et al. Comparison between 5-azacytidine treatment and allogeneic stem-cell transplantation in elderly patients with advanced MDS according to donor availability (VidazaAllo study). *J. Clin. Oncol.* **39**, 3318–3327 (2021). 34283629 10.1200/JCO.20.02724

[6] Jillella, A. P. & Ustun, C. What is the optimum number of CD34 + peripheral blood stem cells for an autologous transplant? *Stem Cells Dev.* **13**, 598–606 (2004). 15684827 10.1089/scd.2004.13.598

[7] Wuchter, P. et al. Poor mobilization of hematopoietic stem cells-definitions, incidence, risk factors, and impact on outcome of autologous transplantation. *Biol. Blood Marrow Transpl.* **16**, 490–499 (2010). 10.1016/j.bbmt.2009.11.012 19925876

[8] Aladağ Karakulak, E. et al. CD34 + hematopoietic progenitor cell dose as a predictor of engraftment and survival in multiple myeloma patients undergoing autologous stem cell transplantation. *Turk. J. Med. Sci.* **50**, 1851–1856 (2020). 32512672 10.3906/sag-2001-173 PMC7775700

[9] Curran, M. P. & Goa, K. L. Pegfilgrastim*. Drugs* **62**, 1207–1215 (2002). 12010086 10.2165/00003495-200262080-00012

[10] Kröger, N. et al. A randomized comparison of once versus twice daily recombinant human granulocyte colony-stimulating factor (filgrastim) for stem cell mobilization in healthy donors for allogeneic transplantation. *Br. J. Haematol.* **111**, 761–765 (2000). 11122135

[11] Molineux, G. The design and development of pegfilgrastim (PEG-rmetHuG-CSF, Neulasta®). *Curr. Pharm. Des.* **10**, 1235–1244 (2004). 15078138 10.2174/1381612043452613

[12] Yang, B. B. & Kido, A. Pharmacokinetics and pharmacodynamics of pegfilgrastim. *Clin. Pharmacokinet.* **50**, 295–306 (2011). 21456630 10.2165/11586040-000000000-00000

[13] Pfeil, A. M. et al. Efficacy, effectiveness and safety of long-acting granulocyte colony-stimulating factors for prophylaxis of chemotherapy-induced neutropenia in patients with cancer: a systematic review. *Support Care Cancer*. **23**, 525–545 (2015). 25284721 10.1007/s00520-014-2457-z

[14] Klastersky, J. et al. Management of febrile neutropaenia: ESMO clinical practice guidelines. *Ann. Oncol.* **27**, 111–118 (2016). 10.1093/annonc/mdw325 27664247

[15] Skopec, B., Skerget, M., Zontar, D., Zadnik, V. & Zver, S. Filgrastim-alone versus pegylated filgrastim-alone for autologous peripheral blood stem cells mobilization in newly diagnosed multiple myeloma patients. *Wien Klin. Wochenschr*. **129**, 545–551 (2017). 28439700 10.1007/s00508-017-1205-z

[16] Herbert, K. E. et al. Plerixafor plus pegfilgrastim is a safe, effective mobilization regimen for poor or adequate mobilizers of hematopoietic stem and progenitor cells: a phase I clinical trial. *Bone Marrow Transpl.* **49**, 1056–1062 (2014). 10.1038/bmt.2014.112 24887382

[17] Watts, N. L. et al. Mobilization of hematopoietic progenitor cells for autologous transplantation using pegfilgrastim and plerixafor: efficacy and cost implications. *Biol. Blood Marrow Transpl.* **25**, 233–238 (2019). 10.1016/j.bbmt.2018.09.005 30219699

[18] Goto, H. et al. Feasibility and efficacy of low-dose pegfilgrastim for CD34 + cell mobilization in lymphoma. *J. Clin. Apher*. **35**, 413–419 (2020). 33043486 10.1002/jca.21816

[19] Sutherland, D. R., Anderson, L., Keeney, M., Nayar, R. & Chin-Yee, I. The ISHAGE guidelines for CD34 + cell determination by flow cytometry. International Society of Hematotherapy and Graft Engineering. *J. Hematother*. **5**, 213–226 (1996). 8817388 10.1089/scd.1.1996.5.213

[20] Costa, L. J. et al. Mobilization and transplantation patterns of autologous hematopoietic stem cells in multiple myeloma and non-Hodgkin lymphoma. *Cancer Control*. **22**, 87–94 (2015). 25504282 10.1177/107327481502200111

[21] Tuchman, S. A. et al. Cyclophosphamide-based hematopoietic stem cell mobilization before autologous stem cell transplantation in newly diagnosed multiple myeloma. *J. Clin. Apher*. **30**, 176–182 (2015). 25293363 10.1002/jca.21360 PMC4523145

[22] Uy, G. L. et al. Contribution of chemotherapy mobilization to disease control in multiple myeloma treated with autologous hematopoietic cell transplantation. *Bone Marrow Transpl.* **50**, 1513–1518 (2015). 10.1038/bmt.2015.190 PMC4548821 26301967

[23] Mohty, M. et al. Autologous haematopoietic stem cell mobilization in multiple myeloma and lymphoma patients: a position statement from the European group for blood and marrow transplantation. *Bone Marrow Transpl.* **49**, 865–872 (2014). 10.1038/bmt.2014.39 24686988

[24] Moncada, V., Bolan, C., Yau, Y. Y. & Leitman, S. F. Analysis of PBPC cell yields during large-volume leukapheresis of patients with a poor mobilization response to filgrastim. *Transfusion* **43**, 495–501 (2003). 12662283 10.1046/j.1537-2995.2003.00361.x

[25] Olivieri, A. et al. Proposed definition of ‘poor mobilizer’ in lymphoma and multiple myeloma: an analytic hierarchy process by ad hoc working group Gruppo ItalianoTrapianto Di Midollo Osseo. *Bone Marrow Transpl.* **47**, 342–351 (2012). 10.1038/bmt.2011.82 PMC3296914 21625224

[26] Giralt, S. et al. Optimizing autologous stem cell mobilization strategies to improve patient outcomes: consensus guidelines and recommendations. *Biol. Blood Marrow Transpl.* **20**, 295–308 (2014). 10.1016/j.bbmt.2013.10.013 24141007

[27] Pérez-Simón, J. A. et al. Minimal number of circulating CD34 + cells to ensure successful leukapheresis and engraftment in autologous peripheral blood progenitor cell transplantation. *Transfusion* **38**, 385–391 (1998). 9595022 10.1046/j.1537-2995.1998.38498257378.x

[28] Herbert, K. E. et al. Pegfilgrastim compared with filgrastim for cytokine-alone mobilization of autologous haematopoietic stem and progenitor cells. *Bone Marrow Transpl.* **48**, 351–356 (2013). 10.1038/bmt.2012.145 22858510

[29] Pusic, I. et al. Impact of mobilization and remobilization atrategies on achieving sufficient stem cell yields for autologous transplantation. *Biol. Blood Marrow Transpl.* **14**, 1045–1056 (2008). 10.1016/j.bbmt.2008.07.004 18721768

[30] Isidori, A. et al. Phase II study of a single pegfilgrastim injection as an adjunct to chemotherapy to mobilize stem cells into the peripheral blood of pretreated lymphoma patients. *Haematologica* **90**, 225–231 (2005). 15710576

[31] Kroschinsky, F. et al. Efficacy of single-dose pegfilgrastim after chemotherapy for the mobilization of autologous peripheral blood stem cells in patients with malignant lymphoma or multiple myeloma. *Transfusion* **46**, 1417–1423 (2006). 16934080 10.1111/j.1537-2995.2006.00911.x

[32] Neumann, T. A. Foote, M. The safety profile of filgrastim and pegfilgrastim. *Twenty Years of G-CSF.* 395–408 (2011).

[33] Goto, H. et al. Efficacy and safety of single-dose pegfilgrastim for CD34 + cell mobilization in healthy volunteers: a phase 2 study. *Transplantation* **108**, 996–1003 (2024). 38012835 10.1097/TP.0000000000004880 PMC10962423

[34] Masuda, N. et al. Dose response of pegfilgrastim in Japanese breast cancer patients receiving six cycles of docetaxel, doxorubicin, and cyclophosphamide therapy: a randomized controlled trial. *Support Care Cancer*. **23**, 2891–2898 (2015). 25733000 10.1007/s00520-015-2654-4 PMC4552775

[35] Smith, T. J. et al. American Society of Clinical Oncology. Recommendations for the use of WBC growth factors: American Society of Clinical Oncology Clinical Practice Guideline Update. *J. Clin. Oncol.* **33**, 3199–3212 (2015). 26169616 10.1200/JCO.2015.62.3488

[36] Hosing, C. et al. Fixed-dose single agent pegfilgrastim for peripheral blood progenitor cell mobilization in patients with multiple myeloma. *Br. J. Haematol.* **133**, 533–537 (2006). 16681642 10.1111/j.1365-2141.2006.06054.x

[37] DiPersio, J. F. et al. Phase III prospective randomized double-blind placebo-controlled trial of plerixafor plus granulocyte colony-stimulating factor compared with placebo plus granulocyte colony-stimulating factor for autologous stem-cell mobilization and transplantation for patients with non-Hodgkin’s lymphoma. *J. Clin. Oncol.* **27**, 4767–4773 (2009). 19720922 10.1200/JCO.2008.20.7209

[38] DiPersio, J. F. et al. Plerixafor and G-CSF versus placebo and G-CSF to mobilize hematopoietic stem cells for autologous stem cell transplantation in patients with multiple myeloma. *Blood* **113**, 5720–5726 (2009). 19363221 10.1182/blood-2008-08-174946

[39] Nademanee, A. P. et al. Plerixafor plus granulocyte colony-stimulating factor versus placebo plus granulocyte colony-stimulating factor for mobilization of CD34(+) hematopoietic stem cells in patients with multiple myeloma and low peripheral blood CD34(+) cell count: results of a subset analysis of a randomized trial. *Biol. Blood Marrow Transpl.* **18**, 1564–1572 (2012). 10.1016/j.bbmt.2012.05.017 22683613

[40] Ri, M. et al. Efficacy and safety of plerixafor for the mobilization/collection of peripheral hematopoietic stem cells for autologous transplantation in Japanese patients with multiple myeloma. *Int. J. Hematol.* **106**, 562–572 (2017). 28527129 10.1007/s12185-017-2255-8

[41] Stover, J. T. et al. Evaluation of hematopoietic stem cell mobilization rates with early plerixafor administration for adult stem cell transplantation. *Biol. Blood Marrow Transpl.* **23**, 1290–1294 (2017). 10.1016/j.bbmt.2017.04.007 28411174

[42] Abhyankar, S. et al. A risk-based approach to optimize autologous hematopoietic stem cell (HSC) collection with the use of plerixafor. *Bone Marrow Transpl.* **47**, 483–487 (2012). 10.1038/bmt.2011.133 21725372

[43] Gutiérrez-Aguirre, C. H. et al. Reduced-dose plerixafor as a mobilization strategy in autologous hematopoietic cell transplantation: a proof of concept study. *Transfusion* **59**, 3721–3726 (2019). 31618456 10.1111/trf.15547

[44] Shi, P. A. et al. Plerixafor mobilization kinetics up to 18 hours. *Transfusion* **54**, 1263–1268 (2014). 24128272 10.1111/trf.12459 PMC3989464

[45] Micallef, I. N. et al. Cost-effectiveness analysis of a risk-adapted algorithm of plerixafor use for autologous peripheral blood stem cell mobilization. *Biol. Blood Marrow Transpl.* **19**, 87–93 (2013). 10.1016/j.bbmt.2012.08.010 22922211

