# Supplementary material for: Impact of single dose of pegfilgrastim on peripheral blood stem cell harvest in patients with multiple myeloma or malignant lymphoma
Source: Sci Rep. 2025 Apr 25;15:14523. doi: 10.1038/s41598-025-98453-7 (PMC12032086; doi:10.1038/s41598-025-98453-7)
Supplement: Supplementary file 1 — Supplementary Material 1 [file 41598_2025_98453_MOESM1_ESM.docx]

**SUPPLEMENTARY MATERIALS**

**Impact of single dose of pegfilgrastim on peripheral blood stem cell harvest in patients with multiple myeloma or malignant lymphoma**

Hideki Goto, Masashi Sawa, Shin-ichiro Fujiwara, Masaki Ri, Tadao Ishida, Masahiro Takeuchi, Kenji Ishitsuka, Masako Toyosaki, Kazutaka Sunami, Junichi Tsukada, Takashi Sonoki, Aiko Shimogomi, Yuki Ichihashi, Yoshiumi Ouchi, Toshihiro Miyamoto, Masayuki Hino, Yoshinobu Maeda, & Takanori Teshima

**METHODS**

**Study Design**

The screening period was defined as from the date of obtaining consent to the day before the start of the study drug (pegfilgrastim or filgrastim) administration. Potential patients underwent screening and those enrolled entered the mobilization period, which spanned from the administration date (Day 1) to Day 7. The follow-up period was from Day 8 to the examination at the end of the study.

**Data Collection**

Data on physical examination, Eastern Cooperative Oncology Group performance status, investigational drug administration, laboratory tests, vital signs, peripheral blood, and CD34-positive cell counts collected by apheresis were measured at each site.

**Statistical Analysis**

The definitions of the analytical populations of the study were as follows:

1. The full analysis set was the primary population for the efficacy endpoints. This included all patients eligible for enrollment, excluding those who never received the study drug and those whose CD34-positive cell counts collected by apheresis were not tested at any time after administration of the study drug.

2. The per protocol set was established for the purpose of confirming the robustness of the primary endpoint. This included all enrolled patients but excluded patients who did not meet the main eligibility criteria and patients with serious deviations from the study protocol that could affect the primary endpoint analysis.

3. The safety analysis population included all enrolled patients except those who never received any investigational drug.

4. The pharmacokinetic analysis population included all enrolled patients except those who never received the study drug as prescribed and/or who had never had a pharmacokinetic blood draw after the study drug administration

5. The immunogenicity analysis target population included all enrolled patients except those who had never received the study drug as prescribed and patients who had never had a blood draw after the study drug administration to measure serum anti-pegfilgrastim antibody levels.

**Table S1.** Details of the inclusion and exclusion criteria

| Inclusion Criteria |
| --- |
| Patients enrolled in the multiple myeloma (MM) cohort had to meet the following conditions:   - Histologically or pathologically diagnosed MM - Achievement of complete response, stringent complete response, very good partial response, or partial response with induction therapy for newly diagnosed MM |
| Patients enrolled in the malignant lymphoma (ML) cohort had to meet the following conditions:   - Histologically or pathologically diagnosed ML - First or second complete response or partial response |
| Patients enrolled in this study had to meet the following conditions (common in MM and ML cohort):   - Voluntary written informed consent to participation in the study - Age 20–75 years at informed consent - Woman of childbearing potential* and fertile men must agree to use highly effective contraceptive from the time of consent (for women) or from the start of investigational product administration (for men) to 180 days after the last dose of investigational product. Women of childbearing potential must have a negative pregnancy test at screening.   *Women of childbearing potential are defined as all women excluding those who have undergone permanent sterilization, those who are postmenopausal (defined as the absence of menstruation for at least 12 consecutive months without any other medical reason), and those anatomically having no childbearing potential. |
| Exclusion criteria |
| Patients in the MM cohort meeting any of the following criteria were not included in this study.   - Histologically or pathologically diagnosed malignancy other than MM within 2 years before informed consent or histologically or pathologically diagnosed active multiple cancers, except MM. - Completion of chemotherapy within 2 weeks before pegfilgrastim administration or the start of filgrastim administration. However, patients treated with dexamethasone, bortezomib, carfilzomib, ixazomib, or daratumumab may participate in the study regardless of the time of the end of the treatment. - Five or more cycles of prior induction therapy, including lenalidomide. - Three or more cycles of prior induction therapy, including melphalan. |
| Patients in the ML cohort meeting any of the following criteria were not included in this study.   - Histologically or pathologically diagnosed malignancy other than ML within 2 years before informed consent or histologically diagnosed active multiple cancers except ML. - Completion of chemotherapy within 2 weeks before pegfilgrastim administration. |
| Patients meeting any of the following criteria were not included in this study (in either the MM or ML cohort):   - History of allogeneic hematopoietic stem cell transplantation, autologous stem cell transplantation, or chimeric antigen receptor T-cell therapy. - History of any adverse event due to the administration of granulocyte colony-stimulating factor (G-CSF) preparation or apheresis that led to the discontinuation of hematopoietic stem cell collection. - Prior administration of a G-CSF preparation or plerixafor that resulted in failure to collect a sufficient number of hematopoietic cells. - Known hypersensitivity to G-CSF preparations or plerixafor. - Any adverse event due to a previously administered anticancer agent that has not yet returned to Grade 1 or lower per Common Terminology Criteria for Adverse Events version 5.0. However, patients with alopecia, Grade 2 peripheral neuropathy, or laboratory abnormalities only other than those listed in the exclusion criterion about clinical values may participate in the study. - Eastern Cooperative Oncology Group performance status ≥2. - Any of the following criteria were met at screening:   - Absolute neutrophil count: ≤1.5×10^9^/L   - Platelets: ≤100×10^9^/L   - Creatinine clearance (Ccr): ≤50 mL/min   - Aspartate aminotransferase, alanine aminotransferase, or total bilirubin: ≥2.5×Institutional upper limit of normal   Ccr were calculated according to the following formulae:   - - - Male patients: Ccr = {(140-age)×body weight (kg)}/{72×serum creatinine (mg/dL)}     - Female patients: 0.85×{(140-age)×body weight (kg)}/{72×serum creatinine (mg/dL)} - Cardiac or pulmonary conditions judged to be inadequate for apheresis or autologous stem cell transplantation. - Central nervous system invasion, or brain or leptomeningeal metastases - Active infection (including an unexplained fever of ≥38°C), or use of antiviral agents (except for prophylactic administration) within 7 days before pegfilgrastim administration or the start of filgrastim administration - Use of a nitrosourea anticancer agent within 6 weeks before pegfilgrastim administration or the start of filgrastim administration - Hematopoietic stem cell mobilization with a G-CSF preparation within 4 weeks before pegfilgrastim administration or the start of filgrastim administration - History of any surgical intervention such as laparotomy within 4 weeks before pegfilgrastim administration or the start of filgrastim administration. However, patients who underwent mild surgical interventions such as lymph node biopsy may participate in the study. - A positive test result for HIV antibody, hepatitis B surface (HBs) antigen, HBs antibody, antibody, HB core (HBc) antibody, or hepatitis C virus (HCV) antibody at screening. However, patients who met any of the following may participate in the study:   - Positive for HBc antibody and/or HBs antibody on a hepatitis B virus (HBV) test at screening, but HBV-DNA is below the limit of quantification or <20 IU/mL (1.3 LogIU/mL); or HBV-DNA level decreased to below the lower limit of quantification or <20 IU/mL (1.3 LogIU/mL) after the administration of nucleoside analogues (e.g., entecavir and tenofovir) or other drugs   - Positive for HBs antibody alone after hepatitis B vaccination   - Positive for HCV antibody but negative for HCV-RNA at screening - Unable to complete all the assessments and investigational product administration specified in this protocol or unable to follow other study procedures - Use of a G-CSF preparation within 7 days before pegfilgrastim administration or the start of filgrastim administration - Pregnant or breastfeeding female patients - Exposure to other investigational product(s) within 16 weeks before pegfilgrastim administration or the start of filgrastim administration - Any condition judged by the investigator or subinvestigator to be unsuitable for participation in the study |

**Table S2.** CD34-positive cells in PB and cell collection through apheresis. (A) MM cohort, pegfilgrastim group; (B) MM cohort, filgrastim group; (C) ML cohort.

**A** MM cohort, pegfilgrastim group

|  | **Day 4**  ***n* = 29** | **Day 5**  ***n* = 29** | **Day 6**  ***n* = 4** |
| --- | --- | --- | --- |
| Cells in PB (/µL) | 22.7 (3.70–90.0) | 68.8 (12.0–138) | 37.9 (9.20–108) |
| Cell collection (10^6^/kg) | - | 4.29 (0.89–13.8) | 2.13 (1.69–3.57) |
| Collection ratio, *n* (%) | - | 25 (86.2) | 1. (100) |

**B** MM cohort, filgrastim group

|  | **Day 4**  ***n* = 30** | **Day 5**  ***n* = 30** | **Day 6**  ***n* = 2** |
| --- | --- | --- | --- |
| Cells in PB (/µL) | 14.5 (1.30–101) | 72.0 (6.00–171) | 40.2 (5.50–140) |
| Cell collection (10^6^/kg) | - | 4.73 (0.94–13.1) | 0.93 (0.66–1.19) |
| Collection ratio, *n* (%) | - | 29 (96.7) | 1. (96.7) |

**C** ML cohort

|  | **Day 4**  ***n* = 12** | **Day 5**  ***n* = 12** | **Day 6**  ***n* = 4** |
| --- | --- | --- | --- |
| Cells in PB (/µL) | 14.4 (5.30–79.4) | 49.2 (10.0–151) | 29.8 (13.8–134) |
| Cell collection (10^6^/kg) | - | 3.69 (1.11–7.53) | 1.32 (0.81–4.35) |
| Collection ratio, *n* (%) | - | 8 (66.7) | 12 (100) |

Data are median (min–max). ML, malignant lymphoma; MM, multiple myeloma; PB, peripheral blood

**Table S3.** Changes in CD34-positive cell count in peripheral blood.

|  | **MM Cohort** | | **ML Cohort** |
| --- | --- | --- | --- |
|  | **Pegfilgrastim**  **(/µL)** | **Filgrastim**  **(/µL)** | **Pegfilgrastim**  **(/µL)** |
| Day 1 | 1.00 (0.00–6.00) | 1.00 (0.00–5.00) | 1.00 (0.00–2.40) |
| Day 4 | 22.7 (3.70–90.0) | 14.5 (1.30–101) | 14.4 (5.30–79.4) |
| Day 5 | 68.8 (12.0–138) | 72.0 (6.00–171) | 49.2 (10.0–151) |
| Day 6 | 37.9 (9.20–108) | 40.2 (5.50–140) | 29.8 (13.8–134) |

Data are median (min–max). ML, malignant lymphoma; MM, multiple myeloma.

**Table S4.** CD34-positive cells in peripheral blood on Day 5 and collected in first apheresis among who cannot achieve CD34-positive cell counts ≥2×10^6^/kg in a single apheresis.

| **Cohort** | **Group** | **CD34-positive cells in peripheral blood (/µL)** | **CD34-positive cells collected in first apheresis (10^6^/kg)** |
| --- | --- | --- | --- |
| MM cohort | Filgrastim | 6.00 | 0.94 |
| MM cohort | Pegfilgrastim | 12.0 | 0.89 |
| MM cohort | Pegfilgrastim | 116 | 1.67 |
| MM cohort | Pegfilgrastim | 92.5 | 1.12 |
| MM cohort | Pegfilgrastim | 30.0 | 1.13 |
| ML cohort | Pegfilgrastim | 15.8 | 1.58 |
| ML cohort | Pegfilgrastim | 25.7 | 1.98 |
| ML cohort | Pegfilgrastim | 43.8 | 1.87 |
| ML cohort | Pegfilgrastim | 10.0 | 1.11 |

ML, malignant lymphoma; MM, multiple myeloma.

**Table S5.** TEAEs occurring in pegfilgrastim and filgrastim groups during the study period

|  | **MM cohort** | | | **ML cohort** | **Pegfilgrastim**  **total** |
| --- | --- | --- | --- | --- | --- |
|  | **Total**  ***N* = 60** | **Pegfilgrastim**  ***n* = 30** | **Filgrastim**  ***n* = 30** | **Pegfilgrastim**  ***n* = 12** | ***n* = 42** |
|  | ***n* (%)** | ***n* (%)** | ***n* (%)** | ***n* (%)** | ***n* (%)** |
| Patients with any TEAE | 48 (80.0) | 24 (80.0) | 24 (80.0) | 10 (83.3) | 34 (81.0) |
| Back pain | 15 (25.0) | 9 (30.0) | 6 (20.0) | 2 (16.7) | 11 (26.2) |
| Alkaline phosphatase increased | 10 (16.7) | 8 (26.7) | 2 (6.7) | 0 | 8 (19.0) |
| Lactate dehydrogenase increased | 11 (18.3) | 7 (23.3) | 4 (13.3) | 1 (8.3) | 8 (19.0) |
| Headache | 8 (13.3) | 6 (20.0) | 2 (6.7) | 1 (8.3) | 7 (16.7) |
| Hyperuricemia | 7 (11.7) | 5 (16.7) | 2 (6.7) | 0 | 5 (11.9) |
| Pyrexia | 7 (11.7) | 4 (13.3) | 3 (10.0) | 1 (8.3) | 5 (11.9) |
| Diarrhea | 4 (6.7) | 3 (10.0) | 1 (3.3) | 4 (33.3) | 7 (16.7) |
| Bone pain | 6 (10.0) | 3 (10.0) | 3 (10.0) | 2 (16.7) | 5 (11.9) |
| Nausea | 6 (10.0) | 3 (10.0) | 3 (10.0) | 1 (8.3) | 4 (9.5) |
| Malaise | 5 (8.3) | 3 (10.0) | 2 (6.7) | 0 | 3 (7.1) |
| Hypocalcemia | 5 (8.3) | 3 (10.0) | 2 (6.7) | 0 | 3 (7.1) |
| Hypokalemia | 3 (5.0) | 3 (10.0) | 0 | 0 | 3 (7.1) |
| Pain | 4 (6.7) | 2 (6.7) | 2 (6.7) | 1 (8.3) | 3 (7.1) |
| Platelet count decreased | 3 (5.0) | 2 (6.7) | 1 (3.3) | 1 (8.3) | 3 (7.1) |
| Vomiting | 2 (3.3) | 2 (6.7) | 0 | 1 (8.3) | 3 (7.1) |
| Decreased appetite | 3 (5.0) | 1 (3.3) | 2 (6.7) | 1 (8.3) | 2 (4.8) |
| Dizziness | 1 (1.7) | 1 (3.3) | 0 | 1 (8.3) | 2 (4.8) |
| Vascular access site pain | 1 (1.7) | 1 (3.3) | 0 | 0 | 1 (2.4) |
| Tetany | 1 (1.7) | 1 (3.3) | 0 | 0 | 1 (2.4) |
| Arthralgia | 1 (1.7) | 1 (3.3) | 0 | 0 | 1 (2.4) |
| Pain in extremity | 1 (1.7) | 1 (3.3) | 0 | 0 | 1 (2.4) |
| Facial spasm | 1 (1.7) | 1 (3.3) | 0 | 0 | 1 (2.4) |
| Interstitial lung disease | 1 (1.7) | 1 (3.3) | 0 | 0 | 1 (2.4) |
| Nasal polyps | 1 (1.7) | 1 (3.3) | 0 | 0 | 1 (2.4) |
| Erythema multiforme | 1 (1.7) | 1 (3.3) | 0 | 0 | 1 (2.4) |
| Citrate toxicity | 1 (1.7) | 0 | 1 (3.3) | 1 (8.3) | 1 (2.4) |
| Hepatosplenomegaly | 0 | 0 | 0 | 1 (8.3) | 1 (2.4) |
| Pneumonia fungal | 0 | 0 | 0 | 1 (8.3) | 1 (2.4) |
| Abdominal discomfort | 0 | 0 | 0 | 1 (8.3) | 1 (2.4) |
| Abdominal pain upper | 0 | 0 | 0 | 1 (8.3) | 1 (2.4) |
| Neuropathy peripheral | 2 (3.3) | 0 | 2 (6.7) | 0 | 0 |
| Uric acid increased | 2 (3.3) | 0 | 2 (6.7) | 0 | 0 |
| Constipation | 1 (1.7) | 0 | 1 (3.3) | 0 | 0 |
| Dental caries | 1 (1.7) | 0 | 1 (3.3) | 0 | 0 |
| Vessel puncture site pain | 2 (3.3) | 0 | 2 (6.7) | 1 (8.3) | 1 (2.4) |
| Oral herpes | 1 (1.7) | 0 | 1 (3.3) | 0 | 0 |
| Apheresis related complication | 1 (1.7) | 0 | 1 (3.3) | 0 | 0 |
| Weight decreased | 1 (1.7) | 0 | 1 (3.3) | 0 | 0 |
| Muscle spasms | 1 (1.7) | 0 | 1 (3.3) | 0 | 0 |
| Cancer pain | 1 (1.7) | 0 | 1 (3.3) | 0 | 0 |
| Hypoesthesia | 1 (1.7) | 0 | 1 (3.3) | 0 | 0 |
| Insomnia | 0 | 0 | 0 | 2 (16.7) | 2 (4.8) |
| Flushing | 1 (1.7) | 0 | 1 (3.3) | 0 | 0 |

ML, malignant lymphoma; MM, multiple myeloma; TEAE, treatment-emergent adverse event.

**Table S6.** Summary of laboratory values and vital signs

|  | | **MM cohort** | | | **ML cohort** | **Pegfilgrastim total** |
| --- | --- | --- | --- | --- | --- | --- |
| **Parameter (unit)** | **Visit** | **Total *N* = 60** | **Pegfilgrastim *n* = 30** | **Filgrastim *n* = 30** | **Pegfilgrastim *n* = 12** | ***n* = 42** |
| **Aspartate aminotransferase (U/L)** | **Baseline** | 19.1 ± 6.7 | 20.4 ± 8.3 | 17.8 ± 4.4 | 22.3 ± 6.1 | 21.0 ± 7.7 |
|  | **Day 4** | 18.2 ± 7.4 | 19.4 ± 9.5 | 17.0 ± 4.3 | 23.5 ± 4.0 | 20.5 ± 8.5 |
|  | **Day 5** | 20.9 ± 6.2 | 22.2 ± 6.8 | 19.6 ± 5.4 | 31.3 ± 18.3 | 24.9 ± 11.9 |
|  | **Day 6** | 18.5 ± 6.2 | 18.8 ± 7.2 | 18.0 ± 5.7 | 30.8 ± 4.3 | 24.8 ± 8.5 |
|  | **Day 10** | 18.9 ± 6.2 | 20.6 ± 7.3 | 17.3 ± 4.5 | 21.6 ± 6.5 | 20.9 ± 7.0 |
|  | **End of study** | 18.0 ± 6.6 | 18.8 ± 5.1 | 17.4 ± 7.7 | 16.5 ± 3.5 | 18.5 ± 4.9 |
| **Alanine aminotransferase (U/L)** | **Baseline** | 17.9 ± 8.1 | 18.1 ± 9.4 | 17.8 ± 6.5 | 23.0 ± 13.8 | 19.5 ± 10.9 |
|  | **Day 4** | 14.7 ± 5.4 | 14.6 ± 6.3 | 14.9 ± 4.5 | 19.8 ± 10.3 | 16.0 ± 7.9 |
|  | **Day 5** | 15.0 ± 5.5 | 14.5 ± 6.2 | 15.4 ± 4.8 | 25.1 ± 23.8 | 17.6 ± 14.4 |
|  | **Day 6** | 14.5 ± 8.6 | 16.3 ± 10.5 | 11.0 ± 0.0 | 28.5 ± 7.7 | 22.4 ± 10.8 |
|  | **Day 10** | 19.8 ± 8.6 | 21.1 ± 9.8 | 18.6 ± 7.2 | 26.3 ± 13.1 | 22.7 ± 10.9 |
|  | **End of study** | 16.8 ± 7.0 | 17.2 ± 6.7 | 16.4 ± 7.4 | 13.0 ± 4.2 | 16.7 ± 6.5 |
| **Alkaline phosphatase unified (U/L)** | **Baseline** | 88.6 ± 48.9 | 96.1 ± 63.1 | 81.2 ± 27.9 | 81.3 ± 36.2 | 91.8 ± 56.7 |
|  | **Day 4** | 155.8 ± 63.9 | 168.8 ± 77.4 | 142.7 ± 44.3 | 173.5 ± 78.8 | 170.2 ± 76.9 |
|  | **Day 5** | 201.7 ± 79.8 | 218.6 ± 93.7 | 185.3 ± 60.8 | 226.1 ± 120.5 | 220.8 ± 100.7 |
|  | **Day 6** | 266.3 ± 154.4 | 273.0 ± 169.2 | 253.0 ± 181.0 | 161.5 ± 83.7 | 217.3 ± 137.2 |
|  | **Day 10** | 136.1 ± 58.7 | 156.7 ± 68.6 | 115.5 ± 37.9 | 157.7 ± 61.7 | 157.0 ± 65.9 |
|  | **End of study** | 92.9 ± 41.9 | 111.2 ± 52.1 | 78.9 ± 26.0 | 122.0 ± 15.6 | 112.6 ± 48.5 |
| **Platelets (10^9^/L)** | **Baseline** | 262.1 ± 66.9 | 261.8 ± 72.7 | 262.4 ± 61.9 | 238.2 ± 100.1 | 255.0 ± 80.9 |
|  | **Day 2** | 242.3 ± 64.3 | 236.7 ± 68.5 | 248.0 ± 60.5 |  |  |
|  | **Day 3** | 229.7 ± 63.3 | 223.3 ± 68.4 | 236.0 ± 58.3 |  |  |
|  | **Day 4** | 221.0 ± 66.4 | 206.7 ± 66.1 | 235.4 ± 64.5 | 192.0 ± 70.5 | 202.5 ± 66.9 |
|  | **Day 5** | 207.6 ± 61.2 | 196.1 ± 62.9 | 218.6 ± 58.4 | 164.2 ± 56.2 | 186.8 ± 62.1 |
|  | **Day 6** | 142.5 ± 40.9 | 159.5 ± 35.5 | 108.5 ± 33.2 | 84.0 ± 23.3 | 121.8 ± 49.0 |
|  | **Day 10** | 183.8 ± 63.2 | 162.3 ± 47.5 | 205.3 ± 70.2 | 141.9 ± 35.2 | 156.3 ± 44.8 |
|  | **End of study** | 261.7 ± 75.1 | 231.0 ± 43.4 | 285.2 ± 86.4 | 236.5 ± 92.6 | 231.7 ± 47.2 |
| **Lactate dehydrogenase unified (U/L)** | **Baseline** | 219.6 ± 110.2 | 234.9 ± 148.7 | 204.3 ± 45.9 | 205.9 ± 43.6 | 226.6 ± 127.7 |
|  | **Day 4** | 346.9 ± 156.2 | 382.6 ± 191.8 | 311.2 ± 101.2 | 444.8 ± 142.5 | 400.4 ± 179.7 |
|  | **Day 5** | 439.9 ± 146.1 | 487.2 ± 162.4 | 394.3 ± 113.2 | 590.1 ± 215.1 | 517.3 ± 182.9 |
|  | **Day 6** | 417.0 ± 177.5 | 461.8 ± 190.0 | 327.5 ± 159.1 | 532.8 ± 216.6 | 497.3 ± 192.4 |
|  | **Day 10** | 226.4 ± 51.0 | 247.4 ± 54.1 | 205.4 ± 38.2 | 278.1 ± 81.3 | 256.4 ± 63.8 |
|  | **End of study** | 180.0 ± 28.3 | 181.5 ± 30.0 | 178.8 ± 27.9 | 185.0 ± 17.0 | 181.9 ± 28.2 |
| **Weight (kg)** | **Baseline** | 63.6 ± 12.5 | 62.9 ± 11.7 | 64.3 ± 13.4 | 65.1 ± 12.0 | 63.5 ± 11.6 |
| **Systolic blood pressure (mmHg)** | **Baseline** | 129.8 ± 17.2 | 126.8 ± 17.1 | 132.7 ± 17.0 | 116.1 ± 7.8 | 123.7 ± 15.7 |
|  | **Day 4** | 123.3 ± 15.2 | 121.0 ± 14.6 | 125.7 ± 15.6 | 110.9 ± 10.5 | 118.1 ± 14.2 |
|  | **Day 5** | 120.0 ± 17.0 | 116.0 ± 14.5 | 124.0 ± 18.6 | 112.6 ± 13.5 | 115.0 ± 14.2 |
|  | **Day 6** | 115.7 ± 9.3 | 111.0 ± 7.5 | 125.0 ± 0.0 | 111.5 ± 7.3 | 111.3 ± 6.9 |
|  | **Day 10** | 129.5 ± 16.5 | 128.4 ± 19.2 | 130.6 ± 13.7 | 116.8 ± 12.7 | 125.0 ± 18.2 |
|  | **End of study** | 130.6 ± 16.2 | 123.5 ± 18.0 | 136.1 ± 12.6 | 119.0 ± 11.3 | 122.9 ± 17.0 |
| **Diastolic blood pressure (mmHg)** | **Baseline** | 82.1 ± 11.2 | 80.7 ± 10.8 | 83.4 ± 11.7 | 70.0 ± 9.5 | 77.6 ± 11.4 |
|  | **Day 4** | 74.5 ± 11.1 | 73.5 ± 10.9 | 75.4 ± 11.5 | 68.1 ± 7.9 | 72.0 ± 10.3 |
|  | **Day 5** | 71.9 ± 11.0 | 72.0 ± 10.7 | 71.8 ± 11.4 | 69.8 ± 11.9 | 71.3 ± 11.0 |
|  | **Day 6** | 71.7 ± 5.3 | 74.3 ± 4.0 | 66.5 ± 3.5 | 76.0 ± 9.4 | 75.1 ± 6.8 |
|  | **Day 10** | 80.2 ± 12.4 | 79.6 ± 11.3 | 80.9 ± 13.5 | 74.2 ± 9.8 | 78.0 ± 11.0 |
|  | **End of study** | 82.6 ± 12.0 | 80.8 ± 11.1 | 83.9 ± 12.8 | 68.5 ± 9.2 | 79.2 ± 11.4 |
| **Pulse rate (beats/min)** | **Baseline** | 76.4 ± 14.5 | 75.5 ± 14.6 | 77.3 ± 14.7 | 82.8 ± 11.9 | 77.6 ± 14.1 |
|  | **Day 4** | 81.7 ± 11.3 | 81.1 ± 12.6 | 82.2 ± 10.1 | 89.7 ± 12.2 | 83.6 ± 12.9 |
|  | **Day 5** | 81.5 ± 11.3 | 81.1 ± 11.0 | 82.0 ± 11.8 | 87.7 ± 11.1 | 83.0 ± 11.3 |
|  | **Day 6** | 74.0 ± 11.4 | 78.5 ± 9.0 | 65.0 ± 12.7 | 94.8 ± 9.6 | 86.6 ± 12.3 |
|  | **Day 10** | 85.7 ± 14.3 | 87.6 ± 15.3 | 83.9 ± 13.2 | 85.1 ± 9.1 | 86.8 ± 13.7 |
|  | **End of study** | 82.6 ± 10.9 | 81.9 ± 9.3 | 83.2 ± 12.3 | 80.5 ± 3.5 | 81.7 ± 8.7 |
| **Temperature (⁰C)** | **Baseline** | 36.5 ± 0.3 | 36.5 ± 0.3 | 36.5 ± 0.3 | 36.6 ± 0.6 | 36.5 ± 0.4 |
|  | **Day 4** | 36.6 ± 0.3 | 36.7 ± 0.3 | 36.6 ± 0.3 | 36.6 ± 0.5 | 36.6 ± 0.3 |
|  | **Day 5** | 36.6 ± 0.3 | 36.6 ± 0.4 | 36.7 ± 0.3 | 36.8 ± 0.4 | 36.7 ± 0.4 |
|  | **Day 6** | 36.6 ± 0.3 | 36.7 ± 0.3 | 36.4 ± 0.0 | 36.8 ± 0.4 | 36.7 ± 0.3 |
|  | **Day 10** | 36.5 ± 0.3 | 36.6 ± 0.3 | 36.3 ± 0.3 | 36.4 ± 0.6 | 36.5 ± 0.4 |
|  | **End of study** | 36.5 ± 0.3 | 36.6 ± 0.3 | 36.4 ± 0.3 | 36.3 ± 0.4 | 36.6 ± 0.3 |

Data are mean ± SD. ML, malignant lymphoma MM, multiple myeloma; SD, standard deviation.

**Table S7.** TEAEs classified by using plerixafor or not

|  |  | **MM cohort** | | | | | **ML cohort** | | **Pegfilgrastim total** | |
| --- | --- | --- | --- | --- | --- | --- | --- | --- | --- | --- |
| **Pegfilgrastim** | | | **Filgrastim** | | **Pegfilgrastim** | |  | |  |  |
| **+Ple**  ***n* = 15** | **-Ple**  ***n* = 15** | **+Ple**  ***n* = 19** | | **-Ple**  ***n* = 11** | **+Ple**  ***n* = 11** | **-Ple**  ***n* = 1** | **+Ple**  ***n* = 26** | **-Ple**  ***n* = 16** |  |  |
|  | | ***n* (%)** | ***n* (%)** | ***n* (%)** | | ***n* (%)** | ***n* (%)** | ***n* (%)** | ***n* (%)** | ***n* (%)** |
| Patients with any TEAE | | 11 (73.3) | 13 (86.7) | 15 (78.9) | | 9 (81.8) | 9 (81.8) | 1 (100.0) | 20 (76.9) | 14 (87.5) |
| Back pain | | 3 (20.0) | 6 (40.0) | 2 (10.5) | | 4 (36.4) | 2 (18.2) | 0 | 5 (19.2) | 6 (37.5) |
| Alkaline phosphatase increased | | 3 (20.0) | 5 (33.3) | 0 | | 2 (18.2) | 0 | 0 | 3 (11.5) | 5 (31.3) |
| Lactate dehydrogenase increased | | 2 (13.3) | 5 (33.3) | 1 (5.3) | | 3 (27.3) | 1 (9.1) | 0 | 3 (11.5) | 5 (31.3) |
| Headache | | 3 (20.0) | 3 (20.0) | 2 (10.5) | | 0 | 1 (9.1) | 0 | 4 (15.4) | 3 (18.8) |
| Hyperuricemia | | 4 (26.7) | 1 (6.7) | 2 (10.5) | | 0 | 0 | 0 | 4 (15.4) | 1 (6.3) |
| Pyrexia | | 2 (13.3) | 2 (13.3) | 2 (10.5) | | 1 (9.1) | 1 (9.1) | 0 | 3 (11.5) | 2 (12.5) |
| Diarrhea | | 2 (13.3) | 1 (6.7) | 1 (5.3) | | 0 | 4 (36.4) | 0 | 6 (23.1) | 1 (6.3) |
| Bone pain | | 0 | 3 (20.0) | 1 (5.3) | | 2 (18.2) | 1 (9.1) | 1 (100.0) | 1 (3.8) | 4 (25.0) |
| Nausea | | 1 (6.7) | 2 (13.3) | 3 (15.8) | | 0 | 1 (9.1) | 0 | 2 (7.7) | 2 (12.5) |
| Malaise | | 1 (6.7) | 2 (13.3) | 1 (5.3) | | 1 (9.1) | 0 | 0 | 1 (3.8) | 2 (12.5) |
| Hypocalcemia | | 1 (6.7) | 2 (13.3) | 1 (5.3) | | 1 (9.1) | 0 | 0 | 1 (3.8) | 2 (12.5) |
| Hypokalemia | | 2 (13.3) | 1 (6.7) | 0 | | 0 | 0 | 0 | 2 (7.7) | 1 (6.3) |
| Pain | | 2 (13.3) | 0 | 2 (10.5) | | 0 | 1 (9.1) | 0 | 3 (11.5) | 0 |
| Platelet count decreased | | 1 (6.7) | 1 (6.7) | 1 (5.3) | | 0 | 1 (9.1) | 0 | 2 (7.7) | 1 (6.3) |
| Vomiting | | 1 (6.7) | 1 (6.7) | 0 | | 0 | 1 (9.1) | 0 | 2 (7.7) | 1 (6.3) |
| Decreased appetite | | 0 | 1 (6.7) | 0 | | 2 (18.2) | 1 (9.1) | 0 | 1 (3.8) | 1 (6.3) |
| Dizziness | | 0 | 1 (6.7) | 0 | | 0 | 1 (9.1) | 0 | 1 (3.8) | 1 (6.3) |
| Vascular access site pain | | 1 (6.7) | 0 | 0 | | 0 | 0 | 0 | 1 (3.8) | 0 |
| Tetany | | 0 | 1 (6.7) | 0 | | 0 | 0 | 0 | 0 | 1 (6.3) |
| Arthralgia | | 0 | 1 (6.7) | 0 | | 0 | 0 | 0 | 0 | 1 (6.3) |
| Pain in extremity | | 0 | 1 (6.7) | 0 | | 0 | 0 | 0 | 0 | 1 (6.3) |
| Facial spasm | | 0 | 1 (6.7) | 0 | | 0 | 0 | 0 | 0 | 1 (6.3) |
| Interstitial lung disease | | 0 | 1 (6.7) | 0 | | 0 | 0 | 0 | 0 | 1 (6.3) |
| Nasal polyps | | 0 | 1 (6.7) | 0 | | 0 | 0 | 0 | 0 | 1 (6.3) |
| Erythema multiforme | | 0 | 1 (6.7) | 0 | | 0 | 0 | 0 | 0 | 1 (6.3) |
| Citrate toxicity | | 0 | 0 | 1 (5.3) | | 0 | 1 (9.1) | 0 | 1 (3.8) | 0 |
| Hepatosplenomegaly | | 0 | 0 | 0 | | 0 | 1 (9.1) | 0 | 1 (3.8) | 0 |
| Pneumonia fungal | | 0 | 0 | 0 | | 0 | 1 (9.1) | 0 | 1 (3.8) | 0 |
| Abdominal discomfort | | 0 | 0 | 0 | | 0 | 1 (9.1) | 0 | 1 (3.8) | 0 |
| Abdominal pain upper | | 0 | 0 | 0 | | 0 | 1 (9.1) | 0 | 1 (3.8) | 0 |
| Neuropathy peripheral | | 0 | 0 | 0 | | 2 (18.2) | 0 | 0 | 0 | 0 |
| Uric acid increased | | 0 | 0 | 1 (5.3) | | 1 (9.1) | 0 | 0 | 0 | 0 |
| Constipation | | 0 | 0 | 1 (5.3) | | 0 | 0 | 0 | 0 | 0 |
| Dental caries | | 0 | 0 | 0 | | 1 (9.1) | 0 | 0 | 0 | 0 |
| Vessel puncture site pain | | 0 | 0 | 1 (5.3) | | 1 (9.1) | 1 (9.1) | 0 | 1 (3.8) | 0 |
| Oral herpes | | 0 | 0 | 0 | | 1 (9.1) | 0 | 0 | 0 | 0 |
| Apheresis related complication | | 0 | 0 | 0 | | 1 (9.1) | 0 | 0 | 0 | 0 |
| Weight decreased | | 0 | 0 | 0 | | 1 (9.1) | 0 | 0 | 0 | 0 |
| Muscle spasms | | 0 | 0 | 0 | | 1 (9.1) | 0 | 0 | 0 | 0 |
| Cancer pain | | 0 | 0 | 0 | | 1 (9.1) | 0 | 0 | 0 | 0 |
| Hypoesthesia | | 0 | 0 | 0 | | 1 (9.1) | 0 | 0 | 0 | 0 |
| Insomnia | | 0 | 0 | 0 | | 0 | 2 (18.2) | 0 | 2 (7.7) | 0 |
| Flushing | | 0 | 0 | 1 (5.3) | | 0 | 0 | 0 | 0 | 0 |

ML, malignant lymphoma; MM, multiple myeloma; TEAE, treatment-emergent adverse event; -Ple, patients without plerixafor; +Ple, patients with plerixafor.


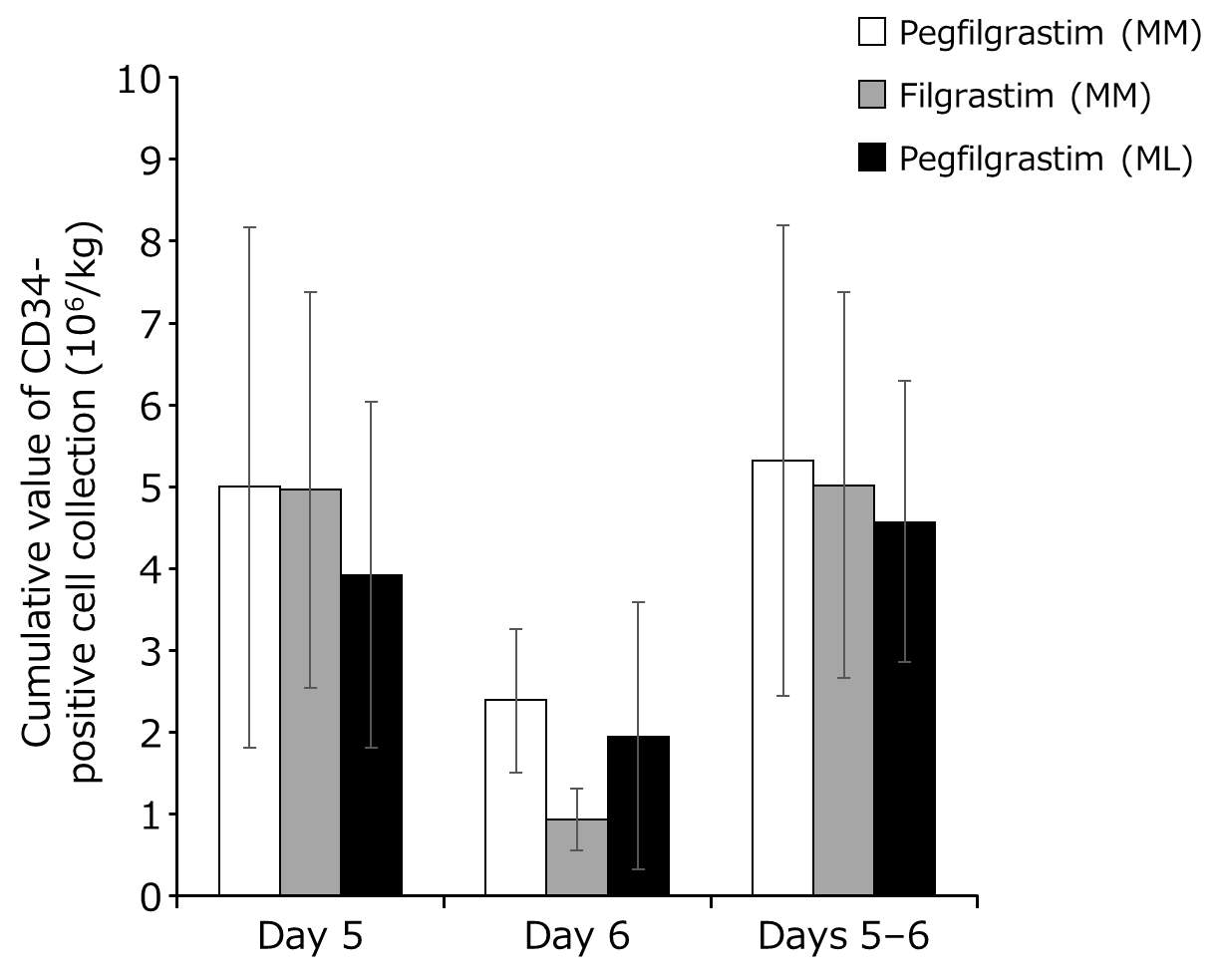


**Figure S1.** Mean CD34-positive cell collection during apheresis. Error bars indicate SD. ML, malignant lymphoma MM, multiple myeloma


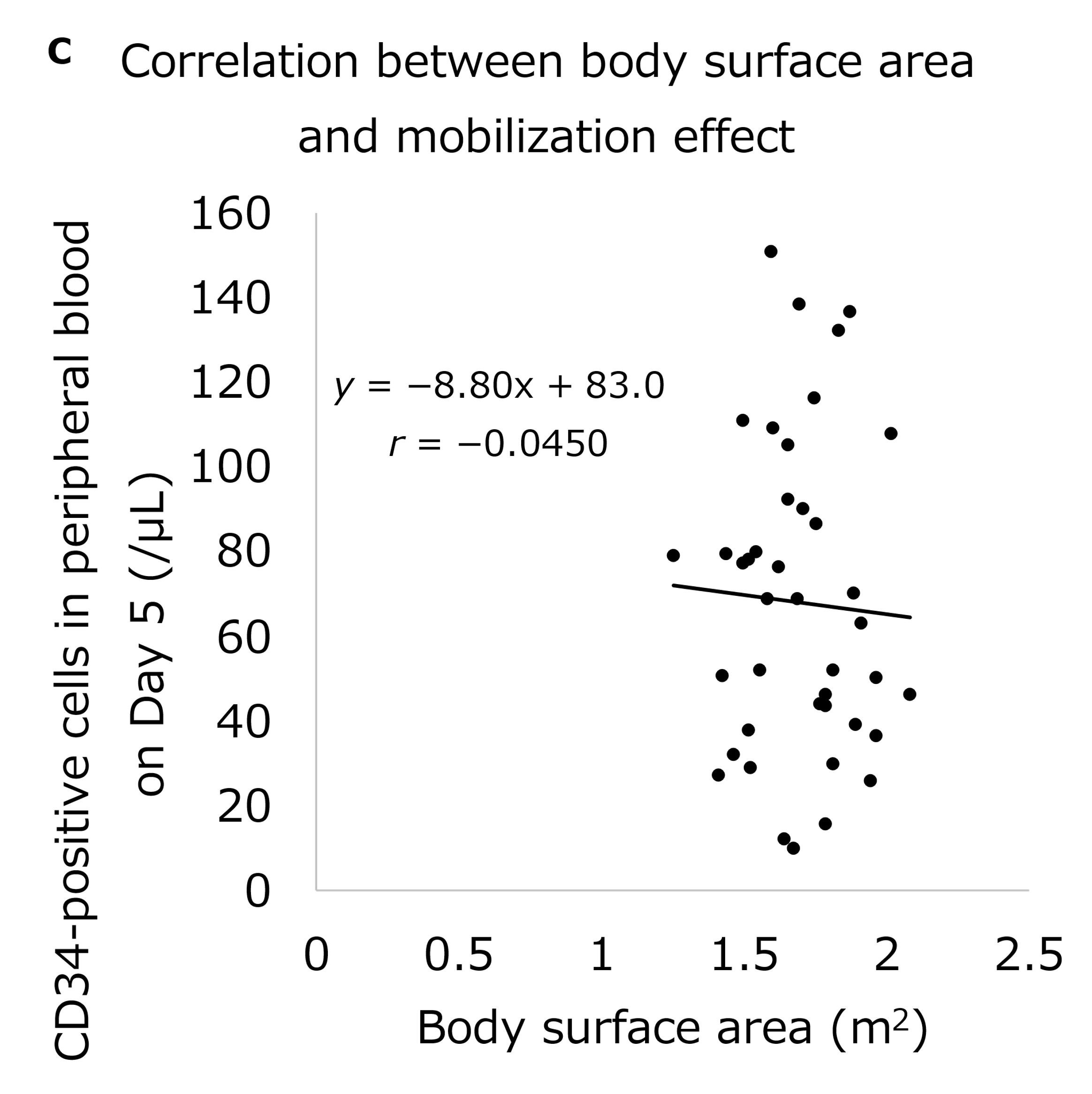

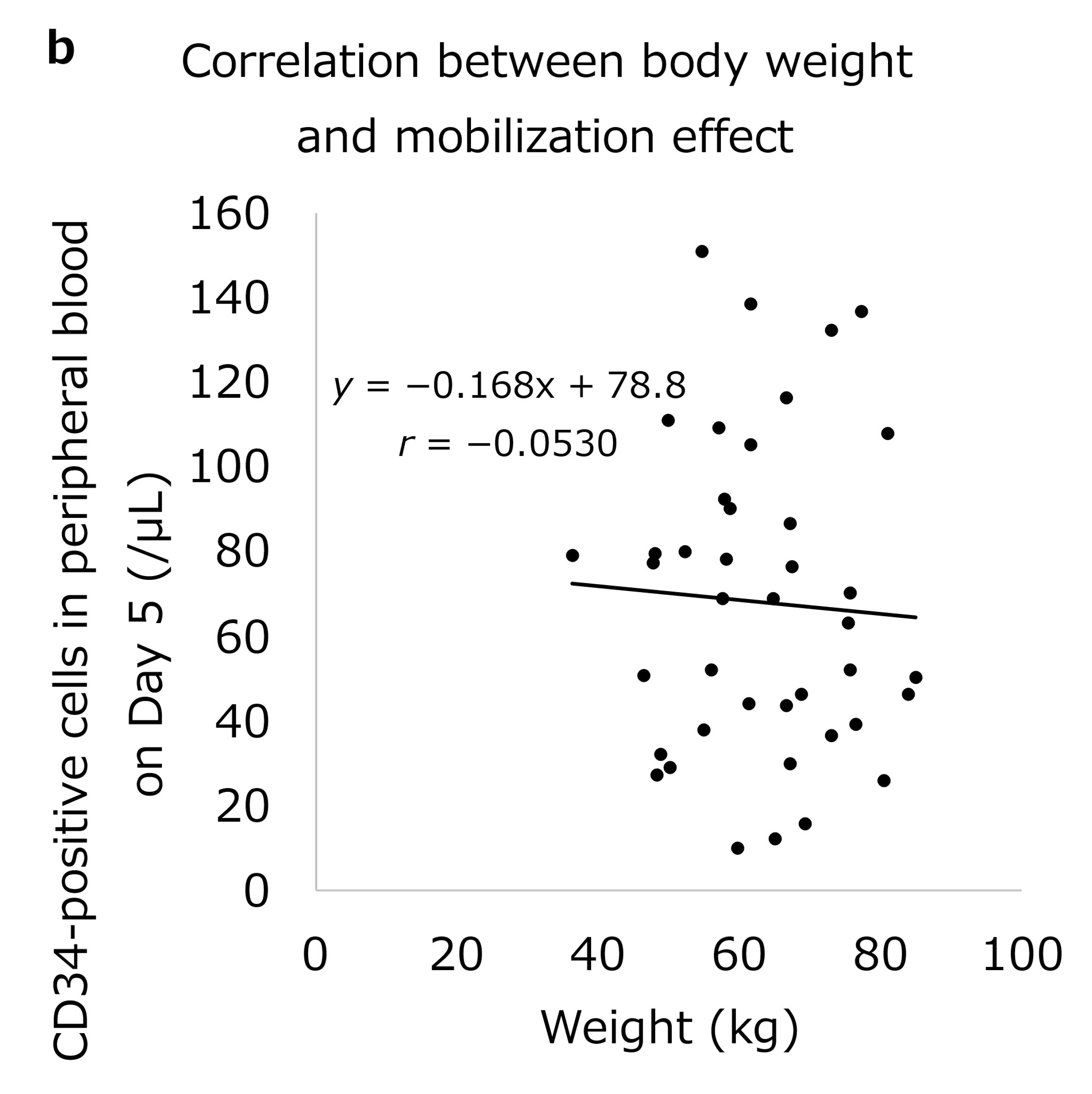

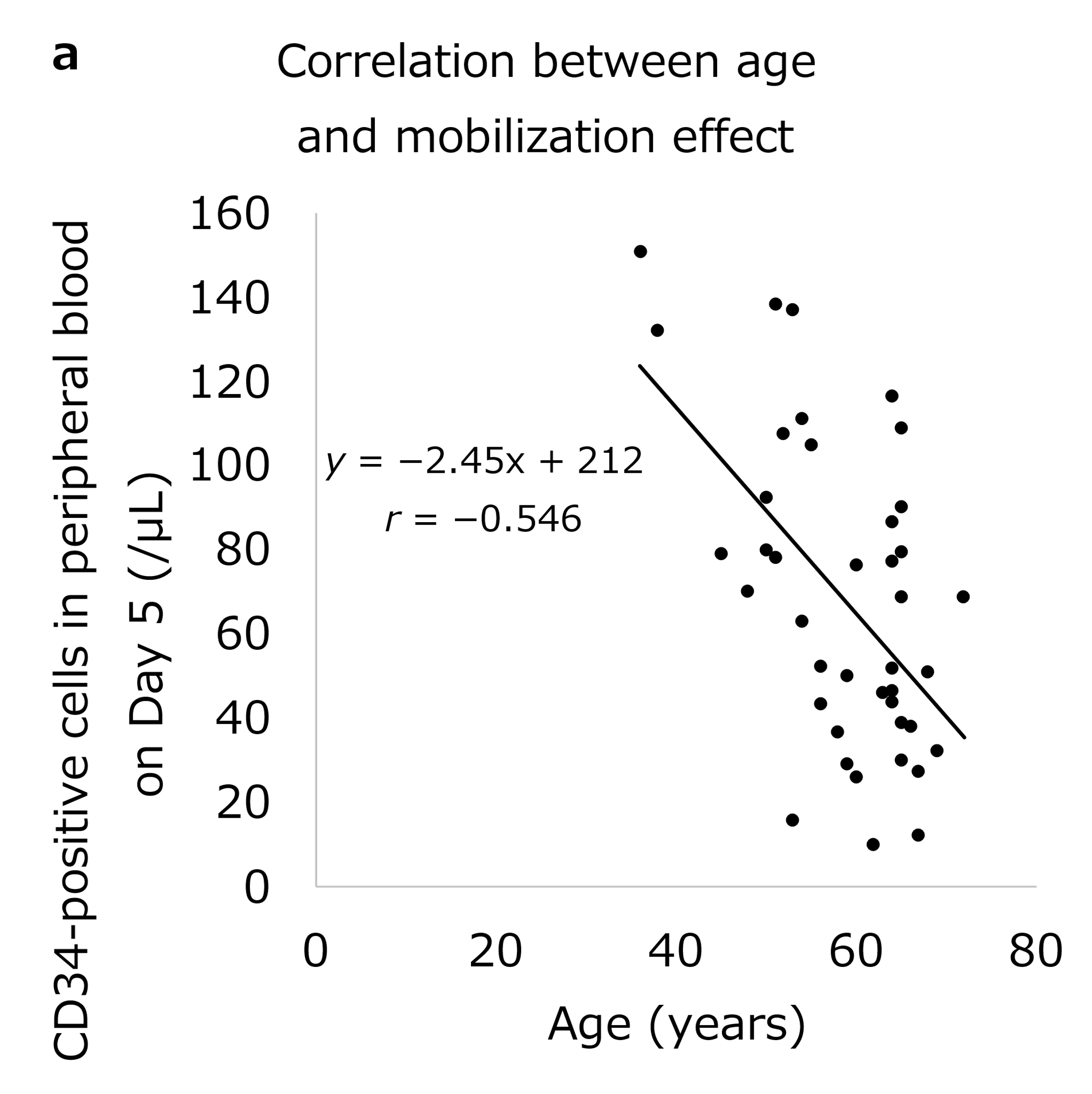


**Figure S2.** Correlation between CD34-positive cells in peripheral blood on Day 5 and age, **a**, body weight, **b**, or body surface area, **c**, in patients receiving pegfilgrastim.
